# Deliberate enhancement of rainfall using desert plantations

**DOI:** 10.1073/pnas.1904754116

**Published:** 2019-09-03

**Authors:** Oliver Branch, Volker Wulfmeyer

**Affiliations:** ^a^Institute of Physics and Meteorology, University of Hohenheim, 70593 Stuttgart, Germany

**Keywords:** desert plantations, weather modification, rainfall enhancement

## Abstract

Large-scale afforestation is increasingly being considered as a negative emissions method for sequestering large quantities of atmospheric CO_2_. At the same time, regional weather modification methods, like cloud seeding, are being used to counteract increasing water scarcity in arid regions. Large-scale sustainable desert agroforestry plantations can contribute to climate change mitigation and can also be used to modify regional climate, particularly rainfall. Climate impacts from plantations need to be well understood before considering implementation. Typically, impact studies are attempted at continental or global scales and use coarse-resolution models, which suffer from severe systematic errors. This is highly problematic because decision makers should only countenance geoengineering schemes like global afforestation if impacts are understood on the regional scale. We posit the necessity of using high-resolution regional models with sophisticated representations of land–atmosphere feedback and vegetation. This approach allows for studying desert plantations and the process chain leading to climate modification. We demonstrate that large-scale plantations enhance regional clouds and rainfall and derive an index for predicting plantation impacts. Thus, desert plantations represent a unique environmental solution via predictable regional weather modification and carbon storage.

## Significance

Our desert plantation concept aligns closely with research into biological carbon sequestration solutions but uniquely extends into the purview of deliberate rainfall enhancement. With this synergy of carbon sequestration and regional weather modification, we can counteract water scarcity and desertification while minimizing conflicts with food croplands. We have demonstrated that large plantations do enhance rainfall in arid regions and identified the underlying process chain. By using this knowledge we have developed a global index to assess which deserts are most favorable for weather modification and discuss how rainfall impacts can be intensified using agricultural methods. This potential for rainfall enhancement and carbon sequestration makes the research extremely interesting for the scientific community and for society.

At the 2015 COP-21 (Conference of the Parties) summit in Paris, a political consensus was reached that countries should take actions to limit the global mean temperature rise to “well below 2 °C.” Conviction is growing that in order to meet this target, emission reductions will need to be supplemented by negative emission technologies (NETs) (1). An important role could be played by afforestation and forest protection schemes (2), which are already the subject of several UN programs (e.g., www.un-redd.org). Afforestation is known as a biological capture-biological storage method (BCBS), distinguishable from geological storage methods like bioenergy and carbon capture with storage (3). When implemented on large enough scales to contribute to a NET strategy, afforestation would go beyond regional climate adaptation and be considered as geoengineering (4–6).

When proposing CO_2_ removal methods such as BCBS, it is fundamental to consider land conflicts with food production and natural landscapes (1, 7). Unsustainable trade-offs might largely be avoided by exclusive afforestation of marginal arid regions, with plantations of hardy crops—hereby named desert agroforestry. Starting with coastal deserts, a “greening” of up to 1 × 10^9^ ha may be possible (8). Related projects are the Bonn Challenge (9), the Great Green Wall for Africa (10), and the Three-North Shelter Forest Program in China (11). Desert plantation potentials include significant carbon storage in vegetation and soils and qualification for carbon trading schemes (12), provision of valuable seed oils and bioenergy (13–15), increased viability of dryland agriculture (16), and soil protection and reversal of desertification (17). There are inherent problems with desert agroforestry though, primarily due to rainfall scarcity, poor soils, and high cost (18). These challenges can be overcome using specialist crop systems, efficiently irrigated (19) with desalinated–urban-waste water (20, 21), powered by solar and bioenergies (8). Recent studies posit the increasing feasibility and sustainability of large-scale desert plantations (8, 22), with the first study estimating that a 100 × 100 km *Jatropha curcas* plantation system would accumulate carbon at 17 to 25 Gkg C y^−1^. This is a substantial quantity, even approaching the CO_2_ emissions of some nation states. The Intergovernmental Panel on Climate Change (1) estimates that a best-case climate trajectory would require a negative emission of 500 to 3,000 Gkg C y^−1^ and at the worst case, 7,000 to 11,000 Gkg C y^−1^. Several plantations could contribute significantly to those rates.

To our knowledge, impact studies of land use change rarely consider local and downstream changes of weather, land–atmosphere (L–A) feedback processes, diurnal cycles, or teleconnections, and coarse-scale global models are too often applied. With their coarser grid scales, limited representation of land surface heterogeneity, and use of convection parameterization, they are acknowledged to be inferior to finer-scale models, especially with respect to simulating energy and water cycles at lower mesoscales (23).

The resulting uncertainties are a matter of concern because national decision makers can only countenance large-scale desert agroforestry if the regional climate impacts are well understood and preferably beneficial. Expected impacts include local changes of temperature, winds, and precipitation (24–28). An understanding of these processes with respect to specific agroforestry scenarios is essential and will provide insight into the possibility of engineering more favorable climates with plantations. We call this biogeoengineering.

In this work, we investigate the impacts of desert agroforestry, using high-resolution models with sophisticated representations of the land surface. Thus, we applied the Weather Research and Forecasting (WRF) coupled to the land model Noah over Oman and Israel to simulate the impact of desert plantations (8, 29, 30). The results provide a strong basis for understanding the complex process chain leading to regional climate modification and potentially, larger-scale impacts.

We demonstrate that large plantations trigger a process chain leading to wind convergence and an increase in clouds and precipitation, under unstable large-scale conditions. Based on our new process understanding, along with statistical analysis, we have derived a Global Feedback Index (GFI) for predicting plantation impacts on regional climate. We propose to apply this GFI to optimize the location of plantations. In this way we can increase rainfall enhancement through judicious selection of arid regions, to maximize sustainability and climate change mitigation potential.

## Materials and Methods

**Fig. 1.**
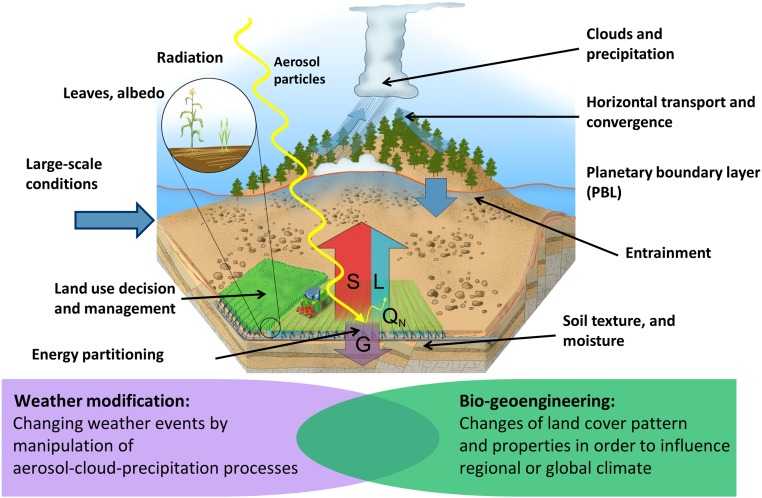
Key processes and feedbacks to be considered for a realistic simulation of desert agroforestry.

**Fig. 2.**
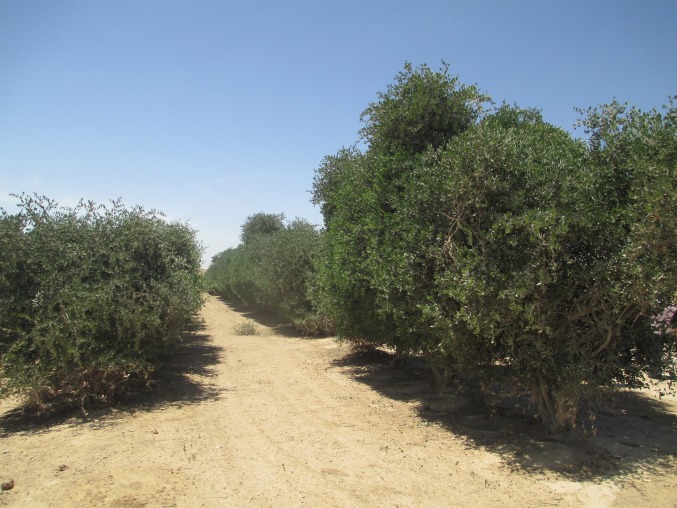
*Jojoba chinensis* plantation with subsurface irrigation at Kibbutz Hatzerim, Beer’sheva, Israel.

Our goal was to simulate the climate impacts of a 100 × 100 km plantation in 2 arid regions, Oman and Israel. For such an impact study, it is vital to simulate plants, irrigation, and soils correctly because atmospheric convection is tightly coupled with surface exchanges of heat, moisture, and momentum (31), which are controlled by land surface properties and water availability. Key processes which must be accounted for are depicted in Fig. 1. Accordingly, we adapted the WRF-Noah model to ensure a representative simulation, using the hardy desert jojoba shrub (*Simmondsia chinensis*) for the plantations (Fig. 2). Key adaptations include a subsurface irrigation scheme, new parameter sets for simulating the jojoba plant, and the judicious selection of physics parameterization schemes (*SI Appendix*, *Methods*). Important parameters, such as jojoba stomatal resistance and albedo, were derived from a purpose-designed study including a jojoba model simulation and validation (30). The study also demonstrated an excellent performance of the WRF-Noah model chain and associated physics schemes when compared with in situ measurements.

Simulations were run at a high horizontal grid increment of 2 km, covering a 400 × 400 km domain, over 71-d from June 21 to August 31, 2012 [previous studies indicate that the strongest impacts occur during summer (7, 23)], and generated 1-h outputs. Simulations were run both with and without plantations (IMPACT and CONTROL, respectively) to allow quantification of impacts and their dependencies on prevailing conditions.

Finally, regional model output from CONTROL and IMPACT were analyzed in terms of precipitation, diurnal cycles of surface energy fluxes, and other atmospheric quantities. More detailed information of the model configuration, simulations, and data analysis are provided in *SI Appendix*, *Methods*.

## Results

### Plantation Impacts.

**Fig. 3.**
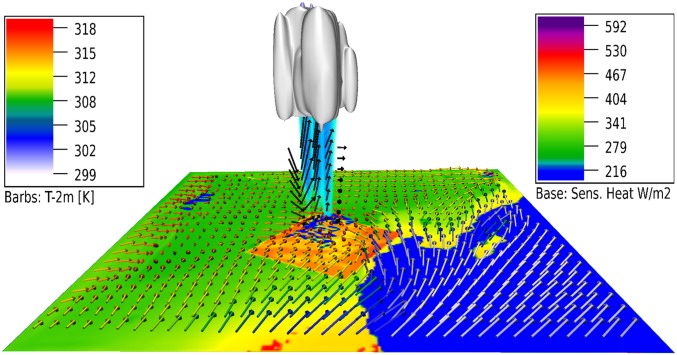
Rainfall impacts over a jojoba plantation in Oman, on June 30 at 14:00 LT. Filled contours are sensible heat in W m^−2^, wind vectors are 10 m winds (unscaled), and vector colors are 2 m temperatures (Kelvin). Black vectors are up/downdrafts. Liquid-phase clouds are gray-silver. Rainfall is blue-turquoise. Fig. 3 was produced with NCAR VAPOR software (see www.vapor.ucar.edu).

Our simulations have yielded profound insights into the L–A feedback process chain over large-scale plantations (Fig. 3). The first step in the chain results from the lower jojoba albedo (surface-reflectance to solar radiation) with respect to the surrounding desert soils (∼17% and 38%, respectively), allowing a greater net surface radiation to be absorbed by the canopy than by the desert (respective typical midday values of ∼700 and ∼400 W m^−2^). This surplus of energy is channeled predominantly into increased upward sensible and latent heat fluxes. These fluxes are further increased by a reduction in downward ground heat flux through canopy shading.

One typically expects cooling rather than heating over liberally irrigated croplands, due to the considerable energy required for evapotranspiration (32). Simulated summer irrigation inputs (June–August) in Israel were low at around 4 mm d^−1^ (seasonal total ∼400 mm), which reflects well canopy observations and local irrigation data (30). This low transpiration allows midday sensible heat to greatly exceed latent heat fluxes (∼450 to 500 and ∼150 to 200 W m^−2^, respectively). This paradox is explained by the high water-use efficiency of plants like jojoba, which can close their stomata while maintaining photosynthetic activity, thus limiting water loss (33). This phenomenon was confirmed by measurements of a midday hiatus in transpiration over desert vegetation (34). The vegetation sensible heating even exceeded that of the surrounding desert by ∼100–150 W m^−2^ (Fig. 3, green to orange gradient). This differential heating (Fig. 3, horizontal vector colors) reduced pressure over the canopy by around ∼1 hPa, leading to wind convergence at the leeside of the plantation (horizontal vectors). Conservation of mass then dictates a steady ascent of surface air. Additionally, the vegetation roughness slows and distorts the winds further, and increases the planetary boundary layer (PBL) depth, thereby lowering the cloud condensation level. It is this combination of convergence and modification of the PBL structure that facilitates convection initiation (35, 36) (seen in Fig. 3 as updrafts, clouds, and precipitation). Interestingly, this phenomenon may be analogous to those occurring in cities, where urban heat island effects are thought to trigger downwind moist convection (37, 38).

In regards to canopy heating, the observed diurnal increase in temperature is compensated for by increased nocturnal cooling through thermal emission losses. Daily mean temperatures are in fact likely to be lower over the plantations than the desert (29, 30).

**Fig. 4.**
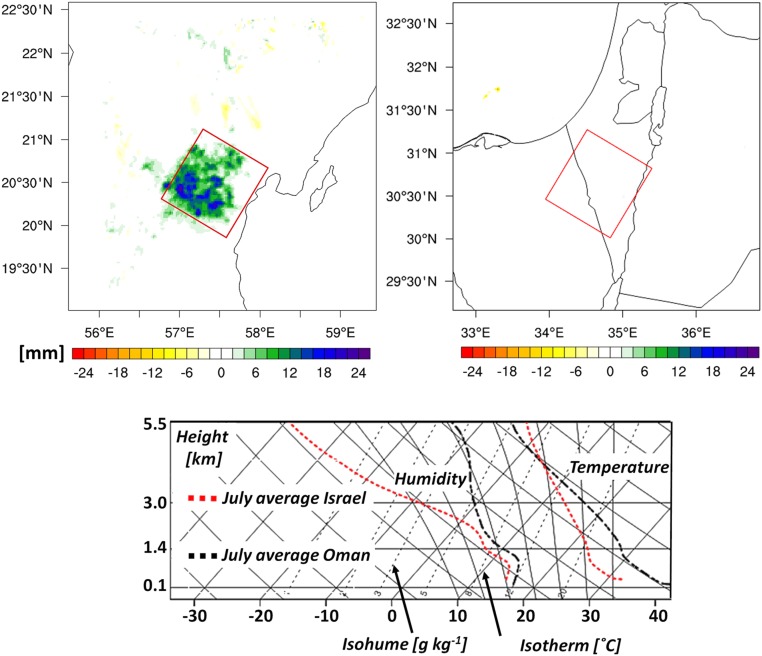
Regional rainfall amounts and atmospheric conditions. (*Top*) Accumulated regional July–August rainfall (71 d; IMPACT) (in millimeters). (*Bottom*) Mean regional skew-T at 12:00 local time, July 2012, averaged over plantation (CONTROL). *Left* is water vapor (in grams per kilograms) and *Right*, virtual temperature (in Celsius).

Convergence and PBL modification are necessary but not sufficient conditions for convection initiation (CI). Upper level conditions must also be unstable enough to support deep convection. In order to study the interactions between surface- and upper-level processes, we examined the accumulated rainfall over 71 d, and the prevailing weather conditions for all 4 scenarios (Fig. 4). In the CONTROL simulations there was very little rainfall—which is typical for summer in Oman and Israel. However, in Oman IMPACT, there was a significant increase in accumulated rainfall (Fig. 4, *Upper Left*)—up to 24 mm in some areas. As a benchmark, the climate station on nearby Masirah Island receives around 25 mm in June and 11 mm in July (National Oceanic and Atmospheric Administrations Monthly Summaries, 1992–2012) (39). Hence, 24 mm represents a significant increase, especially inland where it is even more arid, indicating the potential to significantly reduce irrigation water demand. Intriguingly, there is virtually no impact in Israel over the whole period (Fig. 4, *Upper Right*).

By investigating the prevailing conditions, we have identified the main reasons for this. First, in Israel there was a distinctly lower temperature lapse rate above ∼1.5 km altitude than in Oman (Fig. 4, *Bottom*, 2 right-hand curves), meaning that heated plantation air has to rise to ∼8 km to achieve positive buoyancy, which is unlikely to occur. In Oman, this height was reduced to ∼4 km, which is much more feasible given that desert turbulence often reaches such altitudes. Second, surface temperatures were generally lower in Israel, signifying a more stable lower atmosphere. And third, although humidity in the lower 2 km was relatively similar for both regions, Oman had a more even vertical distribution of moisture above that height (Fig. 4, *Bottom*, 2 left-hand curves). Rising air parcels in Israel would entrain its very dry air, thereby inhibiting saturation. These differences underline that although deserts are arid by definition, regional climate characteristics and causes of aridity may be quite different.

**Fig. 5.**
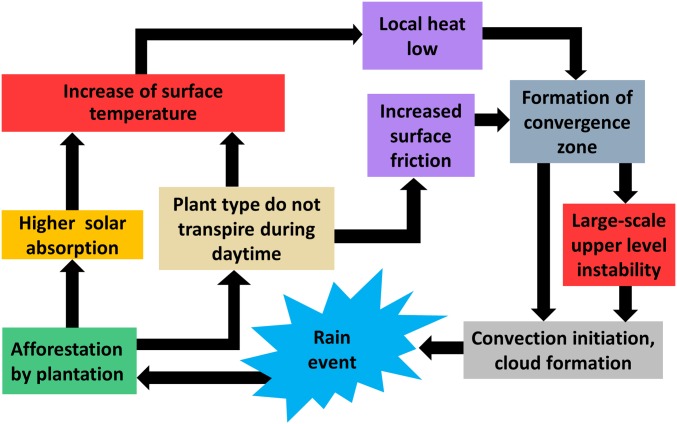
Derived process chain for the understanding of the impact of large-scale plantations in desert regions.

Based on these results, we have derived an understanding of the whole process chain induced by desert agroforestry (depicted schematically in Fig. 5), a prerequisite for realizing large-scale plantations. If plantations are managed so that precipitation amounts are increased, this would represent a win–win situation with respect to water scarcity and climate change mitigation.

### Derivation of a Global Feedback Index.

After identifying necessary climate characteristics for plantation impacts, our aim was to develop a useable metric relating L–A feedback processes with CI for application in global analyses—Global Feedback Index (GFI). Several similar metrics have been developed (40, 41). One—the convection triggering potential (CTP)–low-level humidity deficit (HI_low_) Framework—was developed to identify climate modes in which convection is more or less likely to initiate over dry or wet soils, thus implying where or when L–A coupling is stronger or weaker. The framework includes 2 thermodynamic quantities as markers for these modes—CTP and HI_low_ (41). These variables were later augmented with wind shear dynamics (42), a critical process for CI. Strong wind shear and rapid surface wind speeds can degrade mesoscale circulations induced by patchy landscapes (43, 44). Thus one would expect strong wind speeds and shear to disrupt convergence over plantations. Because of stomatal closure, the plantations exhibit a heating, rather than cooling effect. This together with the lack of direct evaporation from the soil surface means they may be considered similarly to the dry soil category, where a strong influence of the land surface is indicated (41). This is in contrast to wet soil areas where CI tends to be predominantly atmospherically controlled. If statistical relationships between CTP, HI_low_, wind shear, and CI can be identified from the simulations, and suitable thresholds selected, one could theoretically construct a predictive model for plantation impacts.

**Fig. 6.**
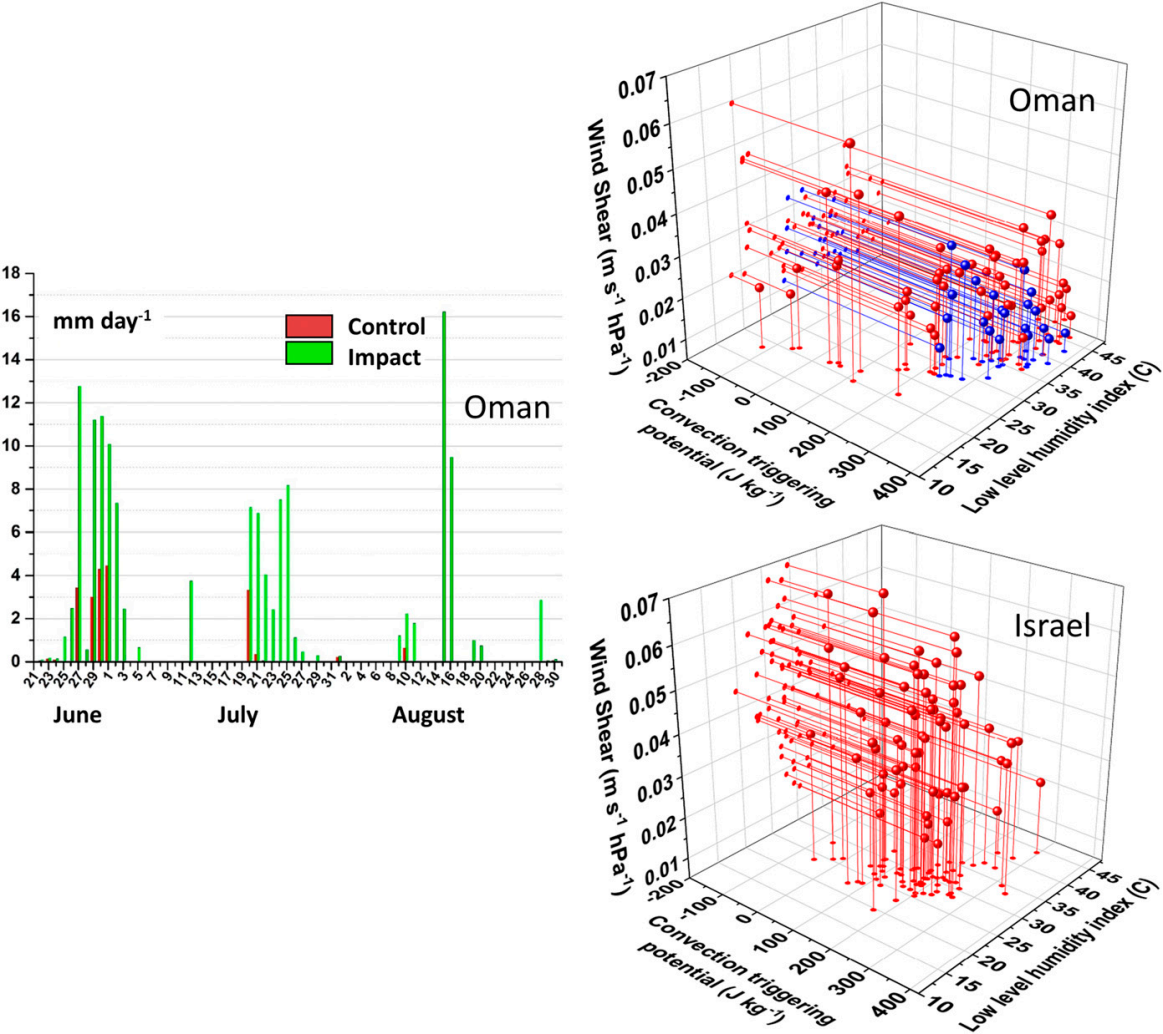
Daily rainfall amounts and atmospheric conditions; (*Left*) daily maximum rainfall over plantation in Oman: red is CONTROL and green is IMPACT (in millimeters per day). (*Right*) Regional CTP–HI_low_–Shear values from CONTROL (plantation areal average), CI days are blue and non-CI are red.

Daily rainfall in Oman is shown in Fig. 6, *Left,* in the form of daily maximum rainfall amounts (for both IMPACT and CONTROL). There are distinct wet and dry periods lasting for several days, almost certainly driven by changing large-scale atmospheric conditions. During the wet periods (e.g., June 25 to July 3), there are days where slight rainfall in CONTROL is increased by IMPACT (June 29 to July 2). On other days, rain falls only within IMPACT (July 21 to 25). This indicates that plantations can intensify naturally occurring rainfall, as well as initiate convection on dry days. We identified 21 different CI days in IMPACT [employing a lower threshold of 1 mm for separating significant rainfall events from model noise (45)].

To assess statistical relationships, we separated and grouped the CI and non-CI days in Oman and Israel and generated within-group statistics for the following 3 variables:

**Variable 1.** CTP (convection triggering potential integrated between 900 and 700 hPa, in Joules per kilogram), calculated as:

$$CTP=g\int_{z=700\;hPa}^{z=900\;hPa}\left(\frac{T_{parcel}-T_{env}}{T_{env}}\right),$$ [1]

where g is gravitational acceleration (in meters per second squared), z is the pressure height (in hectopascals), $T_{parcel}$ is the parcel temperature, and $T_{env}$ is the environmental temperature (in Kelvin).

**Variable 2.** HI_low_, or the sum of dew point depressions at discrete heights of 950 and 850 hPa, in degrees Celsius), calculated as:

$$HI_{low}=\left(T_{950\;hPa}-Td_{950\;hPa}\right)+\left(T_{850\;hPa}-Td_{850\;hPa}\right),$$ [2]

where T is temperature and Td is dewpoint temperature (in Kelvin).

**Variable 3.** Shear, wind speed shear between discrete heights of 850 and 700 hPa, in meters per second per hectopascal).

These variables were calculated as an average between 07:00 and 09:00 AM local time to characterize the morning preconvective environment. Daily values for CTP–HI_low_–Shear for Oman (and Israel) are shown in the right-hand panels (Fig. 6, *Top* and *Bottom*, respectively) for CI (blue) and non-CI days (red).

For all variables, there were statistically significant differences in means (*t* test, *P* < 0.05). CI tends to occur on days with higher CTP values [302.70 J kg^−1^ (±46.98 SD (σ))] than non-CI days [218.42 J kg^−1^ (±112.30)]. For HI_low_, CI days occurred within the distribution of 38.01 °C (±4.30) and non-CI days, 33.15 °C (±6.69). CI days occurred with lower wind shear [0.018 m s^−1^ hPa^−1^ (±0.009)] than non-CI days [0.033 m s^−1^ hPa^−1^ (±0.014)].

These findings are more or less consistent with expectations. CTP provides the energy needed for CI, and so higher CTP permits CI more easily. Conversely, low wind shear permits circulations to develop more easily. For HI_low_, a midrange is optimal for CI. Dependent on moisture and temperature, the midrange indicates that some convective inhibition is present (perhaps due to a temperature inversion), but not so much that it cannot be broken down by surface heating.

In order to develop our GFI, we selected thresholds for the 3 variables, based on the in-group Israel and Oman statistics from the WRF simulations. For each variable, we awarded a score of (1, optimal; 0.5, semioptimal; or 0, suboptimal) depending on how closely values met the conditions observed on CI days, with an emphasis on remaining conservative.

**CTP** was scored thus: 1 (when CTP is greater than CI mean), 0.5 (CTP greater than 0.5σ below CI mean), and 0 (CTP less than 0.5σ below CI mean).
**Shear** was scored: 1 (Shear less than CI mean), 0.5 (Shear less than 0.5σ above CI mean), and 0 (Shear higher than 0.5σ above CI mean).
**HI**_**low**_ was scored using a range: 1 (HI_low_ within CI mean ± 0.5σ), 0.5 (HI_low_ outside CI mean ± 0.5σ, but inside CI mean ± 1σ), and 0 (HI_low_ outside mean ± 1σ).

### Global Analysis of Arid Regions.

The objective was to employ the Global Feedback Index in conjunction with global climate data to identify a priori arid zones and seasons with high climate potential for impacts.

**Fig. 7.**
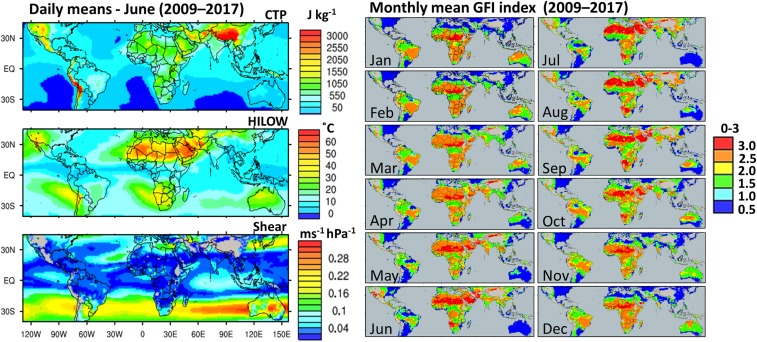
Global analysis of CTP, HI_low_, and Shear from ERA5 data (0.28° resolution). *Left* shows June climatology (2009–2017). *Lower* shows the monthly GFI climatology derived by summing scores (0, 0.5, 1); 3 signifies highest potential and 0, the lowest.

For this we employed the European Centre for Medium-Range Weather Forecasts Re-Analysis 5 (ERA5) global dataset called monthly means of daily means (2009–2017; 0.28° resolution). From this, CTP, HI_low_, and Shear at 06:00 UTC were calculated to apply to the GFI algorithm. We worked on the assumption that values for all 3 variables must be favorable simultaneously, since a suboptimal value for any one variable may disrupt the process chain altogether. Therefore, we consider only those regions with a maximum score of 3 (GFI-3) to be of great interest, or as hotspots. Nevertheless, although regions, or seasons, with GFI-2.5 may indicate suboptimal conditions climatologically, CI might still occur sporadically due to natural variability and are therefore of moderate interest. We consider scores of less than GFI-2.5 to be completely suboptimal. Fig. 7, *Left,* shows the June mean values of CTP, HI_low_, and Shear between 40° S and 45° N. During this month, huge swathes of arid regions can be identified as hotspots (GFI-3). In the Northern Hemisphere, these GFI-3 zones range from Pakistan/Afghanistan/Iran, throughout the Arabian Peninsula (Oman, United Arab Emirates, Saudi Arabia, and Yemen), right across central and south Sahara, and the Sonoran region of southwestern United States/Mexico. In the Southern Hemisphere, the northern Namib desert of Namibia and Angola have GFI-3 in June. Throughout the year (Fig. 7, *Right*), the months between May and October have the highest potential in terms of the amount of land area with GFI-3 (see also *SI Appendix*, Figs. S4–S15 for all monthly data). From November to April, these regions are much reduced, but parts of the Sahara and Arabian Peninsula maintain GFI-3 over the whole year. Reassuringly, the summer GFI-3 climate in Oman and Sonora supports conclusions from preceding plantation simulations, where CI was simulated during summertime (8, 29).

## Discussion and Outlook

We have demonstrated that rainfall enhancement can be realized over desert agroforestry plantations and within a wide range of desert areas—a very exciting prospect for arid or semiarid regions. Increased rainfall can recharge depleted aquifers and significantly reduce irrigation water demand for plantations. We also envisage that the geographical localization of cumulus clouds could also be used to augment other rain enhancement methods, e.g., hygroscopic cloud seeding, which is hampered by the unpredictability of seedable clouds.

We have identified the main physical process chain involved and the primary atmospheric conditions required for impacts, i.e., favorable temperature/humidity profiles and low wind shear. We have also developed a first-order means of assessing suitable regions through global climate data analyses. These assessments can be used to prioritize further studies and to pinpoint final locations for plantations.

We reiterate that, although coarse general circulation model (GCM) simulations of land use change impacts may provide first order insights, sufficient confidence can only be gained using verified regional models. Subdaily temporal resolutions are important for resolving diurnal cycles, and high spatial resolution for good representation of L–A feedbacks, and of complex landscapes. It will also be important to extend investigations to include potential teleconnected effects if very large-scale (e.g., continental) afforestation is considered, perhaps with seasonal latitude-belt simulations (46).

From a management perspective, it should be possible to intensify impacts from the land surface, using agricultural and other geophysical methods, particularly those which minimize transpiration and maximize daytime heating. We know that convergence strength is dependent on horizontal gradients of heating between plantation and surroundings. Conceivably, those gradients can be increased by controlling albedo and evapotranspiration over the vegetation–substrate. For instance, selecting plants with a lower albedo would increase absorbed solar radiation, and thus also the canopy–desert gradient. Correspondingly, selecting areas with high albedo soils would increase the gradient still further. Leaf transpiration reduction could increase sensible heating, achievable through contemporary deficit irrigation techniques like partial root-zone drying (19), which can reduce transpiration while maintaining plant health. Another way would be to withhold irrigation temporarily when optimal weather conditions are observed or forecasted. If the plant stress is manageable, the ensuing leaf stomatal closure would increase heating exactly at the time when impacts are most likely. This method would almost certainly be predicated on using drought-resistant xerophyte species like jojoba (13, 33).

These findings represent a significant first step in the assessment of desert agroforestry as a viable environmental solution from the local to the regional scale. One of the concept’s advantages is its scalability—the ability to start small and scale up over time, which could be a decisive factor in garnering social and political acceptance on the journey toward large-scale implementation. On scales such as those presented here, we have the opportunity to observe regional climate impacts and to assess practical feasibility in terms of energy, water resources, and social and environmental impacts. Successful experimental schemes could then be scaled up to cover multiple regions, with the attendant increase in carbon storage potential. Otherwise, plantations could be maintained at a regional scale, retaining the prospect of controllable rain enhancement. This flexibility, and potential for regional-to-global climate change mitigation, makes desert agroforestry worthy of serious consideration as an environmental solution. In the context of the great need for new negative emissions technologies (1), it should be made a high priority for further research.

## Supplementary Material

Supplementary File

Supplementary File
