# Supplementary material for: Deliberate enhancement of rainfall using desert plantations
Source: Proc Natl Acad Sci U S A. 2019 Sep 3;116(38):18841–7. doi: 10.1073/pnas.1904754116 (PMC6754602; doi:10.1073/pnas.1904754116)
Supplement: Supplementary File [file pnas.1904754116.sapp.pdf]

PNAS
www.pnas.org

Supplementary Information for

Deliberate Enhancement of Rainfall using Desert Plantations

Oliver Branch (first author), Volker Wulfmeyer (co-author)

Email: oliver_branch@uni-hohenheim.de

This PDF file includes:

- Supplementary text
  - Methods
  - Land surface-atmosphere feedbacks
- Figs. S1 to S17
- Tables S1 to S6
- Still of Movie S1
- Caption for Movie S1
- References for SI reference citations

Other supplementary materials for this manuscript include the following:

Movies S1

www.pnas.org/cgi/doi/10.1073/pnas.1904754116

Supplementary Information Text

Methods

Experimental Design

Our first goal was to simulate the weather impacts of a 100 × 100 km rectangular jojoba plantation in two different arid/semi-arid regions - Oman and Israel – in the summer of 2012. Of particular interest are impacts on the PBL and convective processes and rainfall. These impacts were compared against a control run, i.e. without a simulated plantation, to assess changes in 'normal' weather patterns. The second goal was to identify, from these simulations, the necessary conditions for convection initiation, in terms of critical variables for CI, and apply them appropriately within a global analysis of climate reanalysis data. In this way, the aim was to identify promising arid regions and seasons for high rainfall impacts.

Underpinning the whole study is the necessity for a) a good simulated representation of jojoba plantation, ensured by judicious model configuration, sufficiently high resolutions, and high quality forcing and initialisation data, and b) validation with actual in-situ data.

Model configuration and simulations

All model simulations were run with the coupled atmosphere-land surface model, WRF-NOAH[1,2], which provides a full 4-dimensional representation of the land-atmosphere (L-A) system within a specified volume, as driven at the boundaries by weather conditions from the ECMWF-IFS global climate model[3]. See Table S1 for simulation details, Table S2 for the selected physics sub-grid parameterization schemes, and Table 3 for the WRF-NOAH configuration '*namelist'* settings.

Table S1: Details of model simulation showing model type, resolutions, domains and data sources.

Component	Metadata	Reference
**Atmospheric model**	WRF - Weather Research and Forecasting V3.4.1	1
**Grid dimensions**	200 × 200	
**Horizontal resolution**	2 km	
**Vertical levels**	92	
**Vertical resolution**	Varied – sigma terrain-following coordinates	
**Number of soil layers**	4 (10, 30, 60, 100 cm depth)	
**Forcing data**	ECMWF - Operational analysis (0.125˚) 6-hourly European Centre for Medium Range Weather Forecasting	3
**Extra forcing data**	OSTIA sea surface temperatures 12-hourly	UK Met Office/ GHRSST
**Simulation period**	21st June – 31st August 2012	
**Spin up time**	15th – 20th June (7 days, discarded for analysis)	
**Output**	NETCDF – Hourly	

Table S2: Sub-grid physics parameterization schemes selected, along with references for description and developer.

Scheme type	Scheme	Reference
**Land surface model**	NOAH LSM	2
**Boundary layer**	YSU (Yonsei University)	4
**Surface layer**	MM5 Monin-Obhukov	5,6
**Microphysics**	Morrison 2-moment	7
**Shortwave radiation**	RRTMG	8
**Longwave radiation**	RRTMG	9

Table S3: Namelist settings in WRF to specify duration, domain geometry, integration time, physics/dynamics and boundary options.

**Time Control**	**Value**	**Domain**	**Value**	**Physics**	**Value**	**Dynamics**	**Value**
run_days	0,	time_step	12,	mp_physics	10,	w_damping	0,
run_hours	0,	max_dom	1,	ra_lw_physics	4,	diff_opt	1,
start_year	2012,	e_we	200,	ra_sw_physics	4,	km_opt	4,
start_month	01,	e_sn	200,	radt	3,	diff_6th_opt	0,
start_day	01,	e_vert	92,	sf_sfclay_physics	1,	diff_6th_factor	0.12,
start_hour	00,	p_top_requested	5000,	sf_surface_physics	2,	base_temp	290
start_minute	00,	metgrid_levels	92,	bl_pbl_physics	1,	damp_opt	0,
end_year	2012,	num_soil_levels	4,	bldt	0,	zdamp	5000.,
end_month	08,	dx	2000,	cu_physics	0,	dampcoef	0.2,
end_day	31,	dy	2000,	cudt	5,	khdif	0,
end_hour	23,	grid_id	1,	isfflx	1,	kvdif	0,
end_minute	00,	parent_id	1,	ifsnow	1,	non_hydrostatic	.true.,
interval_seconds	21600	i_parent_start	1,	icloud	1,	moist_adv_opt	1,
history_interval	60,	j_parent_start	1,	surface_input_source	1,	scalar_adv_opt	1,
restart	.true.,	parent_grid_ratio	1,	num_soil_layers	4,		
restart_interval	1440,	time_step_ratio	1,	num_land_cat	20,	**Boundaries**	
		feedback	0,	sst_update	1	bdy_width	5,
		smooth_option	0	sf_urban_physics	0,	spec_zone	1,
						relax_zone	4,
						specified	.true.,
						nested	.false.,

We emphasize the importance of using well-tested physics scheme set within WRF, to correctly simulate the process chain operating from the land surface to the troposphere including cloud microphysics. Important studies demonstrating the performance on this study's parameterizations include the YSU boundary layer scheme[10,11,12,13], the Noah LSM[4,14,15,16] and the Morrison microphysics scheme[17,18,19,20].

The scenarios were designed to demonstrate the impact of a 100 × 100 km rectangular jojoba plantation (*Simmondsia chinensis*) on local weather within two different regions: the arid region of Oman and the arid/semi-arid region of Israel (domains and plantations shown in Fig. S1).

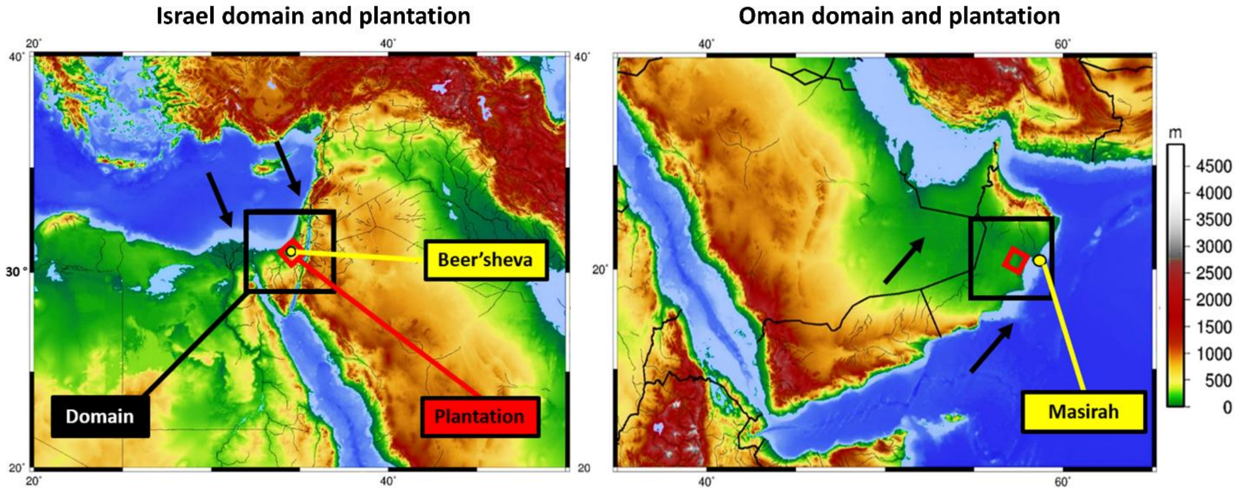

Fig. S1: Model domains and plantations marked on topography in eastern Mediterranean and Arabian Peninsula regions. Model domains are black polygons and plantations are red polygons. Mean summer wind flows are marked with black arrows. The Oman domain is centred at 20.30N, 57.58E and Israel at 31.25N, 34.50E. Both domains are 200×200 in dimension with 2 km horizontal grid spacing. The yellow dots represent the city of Beer'sheva, Israel and Masirah Island, Oman.

Simulations were run both with and without plantations (respectively, IMPACT and CONTROL) over a 71 day period (21st June – 31st August 2012), allowing us to a) quantify modifications of 'normal' or CONTROL weather, and b) assess links between any impacts and the background atmospheric conditions. The plantations were added by inserting a new land use category into the gridded map data used by the model to demarcate different land cover types. Then, unique land-surface characteristics tailored to the jojoba plant were prescribed for plant and parameters in that area (Fig. S1, red polygons). Correct simulation of vegetation, soil and irrigation is critical for any impact study, since convective processes in the atmosphere are tightly coupled with surface exchanges of heat, moisture and momentum at the land surface[21,22] - in turn controlled by soil-vegetation properties and water availability. An updated format of the Harmonized World Soil Database (HWSD) was included to optimize soil texture mapping[23], and plant and soil parameters were selected based on a one year jojoba simulation, and validation with in-situ measurements of jojoba[24]. Important plant parameters were leaf stomatal resistance, albedo, canopy geometry/roughness, and soil properties such as saturated hydraulic conductivity and porosity - obtained from local surveys and literature (See Table S4).

Table S4: Vegetation and soil parameters prescribed for the simulation of the jojoba plantation, based on literature and local in-situ observations. See Branch et al. (2014) for further information on jojoba simulation and validation[24].

Plant and soil variable	Surrounding desert soils	Jojoba plantation
Vegetation roughness length	**0.1 m**	**0.3 m**
Vegetation fraction	**0 %**	**70 %**
Vegetation albedo	**38%**	**12 %**
Vegetation leaf stomatal resistance	**999 s m^{-1}**	**250 s m^{-1}**
Vegetation leaf area index [m^2 m^{-2}]	**0.1**	**3.2**
Soil texture	**Sandy Loam**	**Sandy Loam**
Saturated hydraulic conductivity	**5.23 × 10^{-6} ms^{-1}**	**5.23 × 10^{-6} ms^{-1}**
Soil porosity	**0.38 m^3 m^{-3}**	**0.38 m^3 m^{-3}**
Soil field capacity	**0.31 m^3 m^{-3}**	**0.31 m^3 m^{-3}**

Sub-surface irrigation was also simulated, designed to balance, dynamically, efficient water-use with acceptable plant stress, with a target soil moisture fraction defined by a stress term F_{AW}[25] calculated as:

$$F_{AW} = \frac{\eta - \eta_{wp}}{\eta_{fc} - \eta_{wp}}$$

Where η is soil moisture, η_{wp} is the wilting point, and η_{fc} is the soil field capacity [m^3 m^{-3}]. The resulting minimum necessary soil moisture for sandy-loam, present in the plantation, was 0.18 m^3 m^{-3}. Water was added, when necessary to the second and third model soil layers, i.e. sub-surface, every time step, to maintain this level.

Statistical analysis

Analysis was carried out on the two impact scenarios, by grouping the regional data together and splitting it into CI and non-CI days (See Fig. S2).

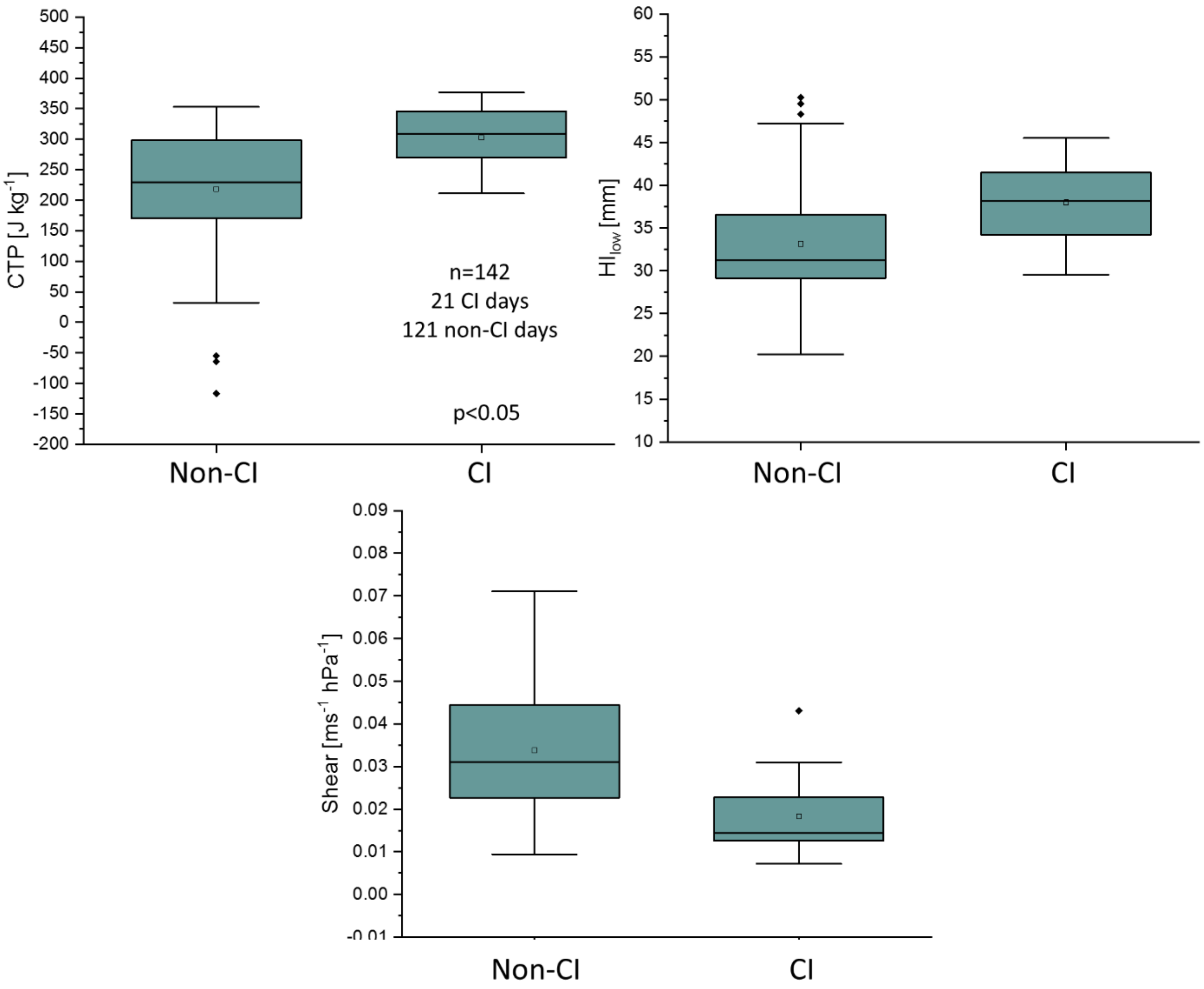

Fig S2. Box plots of CTP, HIlow, Shear and IWV on CI and non-CI days in Israel and Oman (total n=140) using a 2-sample t-test. Means are the central boxes, medians are the central lines, the green box limits are 25-75% limits and whiskers are the 1.5 IQL. Differences in means are statistically different at $p<0.05$, allowing for non-normal distributions (Welch corrected for non-normal distributions).

These days were selected through assessment of whether rainfall occurred or not during the day, using a minimum lower threshold of 1 mm day^{-1} to account for model 'noise'[26]. 21 CI days and 121 non-CI days were identified. These two groups were assessed statistically in terms of four quantities critical for convection initiation. The first is a measure of static stability, dependent on the temperature profile - Convection Triggering Potential between 900 and 700 hPa (CTP, J kg^{-1})[27]. Next is a measure of low-level humidity - the sum of dew point depressions at 950 and 850 hPa (HI_{low}, °C)[27]. Finally a dynamic variable - wind shear between 850 and 700 hPa (Shear, m s^{-1} hPa^{-1})[28].

CTP is calculated as:

$$CTP = g \int_{z=700\ hPa}^{z=900\ hPa} \left(\frac{T_{parcel} - T_{env}}{T_{env}}\right)$$

where g is gravitational acceleration [m s^{-2}], z is the pressure height [hPa], T_{parcel} is the parcel temperature and T_{env} is the environmental temperature [˚K].

HI$_{low}$ is calculated as:

$$HI_{low} = (T_{950\ hPa} - Td_{950\ hPa}) + (T_{850\ hPa} - Td_{850\ hPa})$$

where T is temperature and Td is dewpoint temperature [˚K].

Wind shear was calculated as the difference in wind speed between 850 and 700 hPa [ms^{-1} hPa^{-1}]. All variables were calculated as an average between 07:00 and 09:00 AM local time to characterize the morning pre-convective environment.

Two-sample t-tests were then conducted in Originlab, with a Welch correction to account for non-normal distributions and statistical significance indicated at $p<0.05$. Significant differences in means were observed for all 4 variables (Table S5 and Fig. S2).

Table S5. Grouped mean and standard deviation statistics of 3 key variables for CI and non-CI days, derived from 2-sample t-test (Welch corrected for non-normal distributions).

Variable	CI		Non-CI		
	Mean	SD (σ)	Mean	SD (σ)	t-statistic (non-normal)
**CTP**	302.70	46.98	218.42	112.30	-4.4
**HIlow**	38.01	4.30	33.15	6.69	-4.3
**Shear**	0.018	0.009	0.033	0.014	6.5

Derivation of Global Feedback Index (GFI)

The intention was to assess different arid regions for their climate suitability for weather modification by identifying the ideal conditions for CI, derived from the WRF IMPACT simulations, and applying them to analyses of global climate data. In this way we could identify ideal climate conditions over the Earth's arid regions. For this purpose, we used nine years of monthly means of daily means from the ECMWF ERA5 reanalysis global dataset (2009-2017)[29].

The first task was to identify the ideal conditions for CI, based on the WRF Impact simulations. From Fig. S2, we observed that the mean values of CTP, HI_{low} and Shear on CI days were greater than one standard deviation (1σ) away from the non-CI means (Table 5). CTP optimality increases with increasing values higher values; with no upper bounds. Conversely, Shear optimality decreases with increasing values.

HI_{low} optimality tends to fall into a middle range for the 'dry heating' CI effect, when a high CAPE goes together with moderate CIN that can be overcome by strong heating and convergence. HI_{low} values above this range indicates a very dry atmosphere, which tends to prevent CI altogether. HI_{low} values below this range, indicates high levels of moisture. This condition need not inhibit CI *per se*, but it indicates conditions where the land surface is less coupled with CI, and if CI does

occur, it is more likely to be triggered by larger scale conditions, e.g. when embedded in frontal systems[22]. It is assumed that all quantities need to be optimal simultaneously for CI to occur, because a sub-optimal value for any one variable may disrupt the process chain altogether.

To identify the optimal variable-space for CI, the statistical analyses seen in Table S5 and Fig. S2 were used as a basis. The non-CI day means were statistically different from the CI days (>1σ), and we therefore considered variables as optimal within 1σ around the CI mean. Even then, to be sure of a 'conservative' variable-space we selected even tighter thresholds. Figure S3 shows our score algorithm used for identifying optimal and sub-optimal variable-spaces.

$$\text{CTP} \begin{cases} 0 & CTP < (mean - 0.5\sigma) \\ 0.5 & 0.5\sigma < CTP < mean \\ 1 & CTP > mean \end{cases}$$

$$\text{Hi}_{\text{low}} \begin{cases} 0 & outside\ (mean \pm 1\sigma) \\ 0.5 & outside\ (mean \pm 0.5\sigma), but\ inside\ (mean \pm 1\sigma) \\ 1 & inside\ (mean \pm 0.5\sigma) \end{cases}$$

$$\text{Shear} \begin{cases} 0 & Shear > (mean + 1\sigma) \\ 0.5 & (mean + 0.5\sigma) < Shear < (mean + 1\sigma) \\ 1 & Shear < (mean + 0.5\sigma) \end{cases}$$

Fig S3. Rules for awarding suitability scores for CTP, HI_{low} and Shear using thresholds based on numbers of standard deviations from regional CI mean values. CTP and Shear are threshold variables requiring only lower and upper bounds respectively, and HI_{low} which is optimal within a range, requires upper and lower thresholds.

For CTP we selected values above 0.5σ below the CI mean as being semi-optimal (score 0.5), and values above the CI mean as optimal (score 1), and values below 0.5σ below the mean as suboptimal (score 0). For HI_{low} we assigned scores of 0.5 and 1 to ranges 1σ above and below the mean and 0.5σ above and below the mean, respectively. Values outside 1σ above and below the mean are suboptimal (score 0).

From the ERA5 dataset, we calculated CTP, HI_{low} and generated mean statistics for each month of the year, over the 2009-2017 period and also for the year of simulation, 2012 (Figs. S4-S15). This comparison was made to assess whether the 2012 conditions were representative for the nine year period.

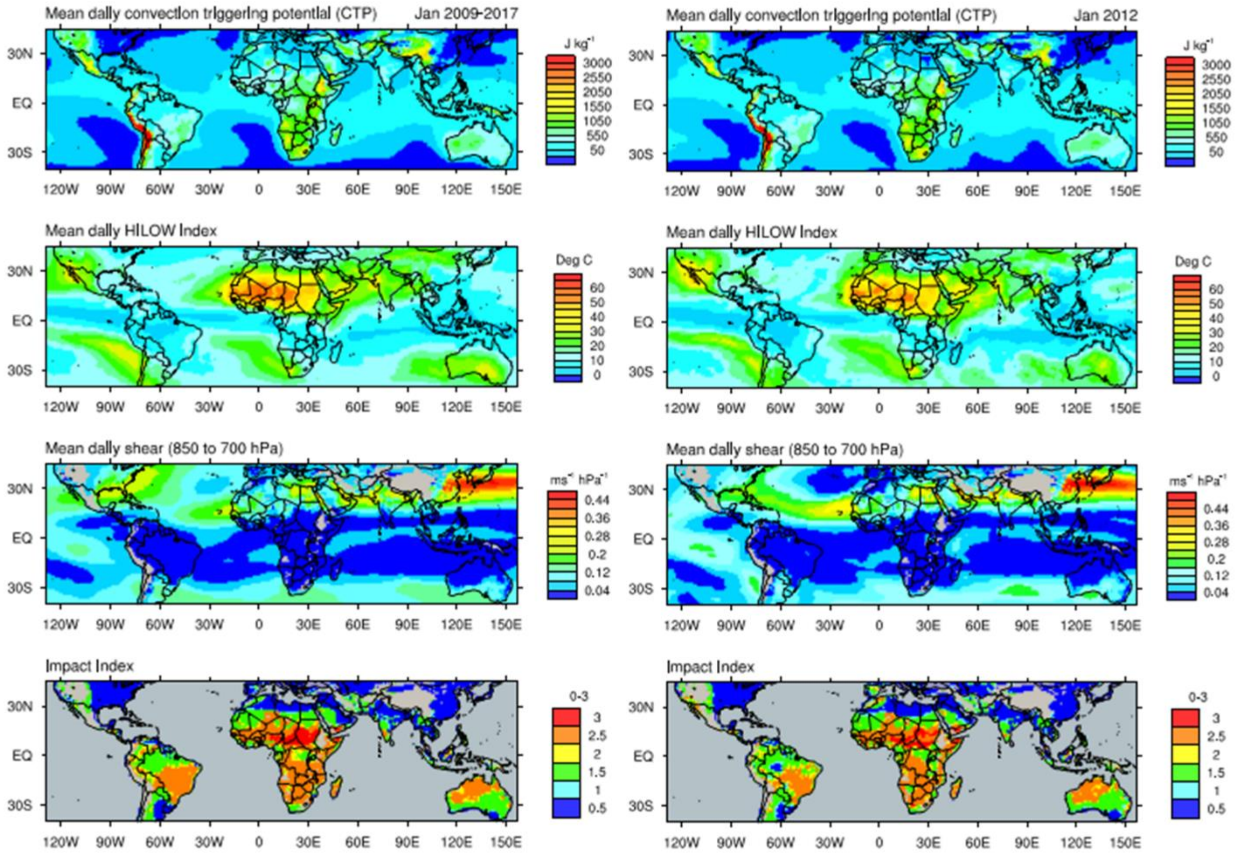

Fig S4. January - monthly mean values of daily means (left panels 2009-2017, right panels 2012 only) for CTP, HIlow, Shear, and GFI.

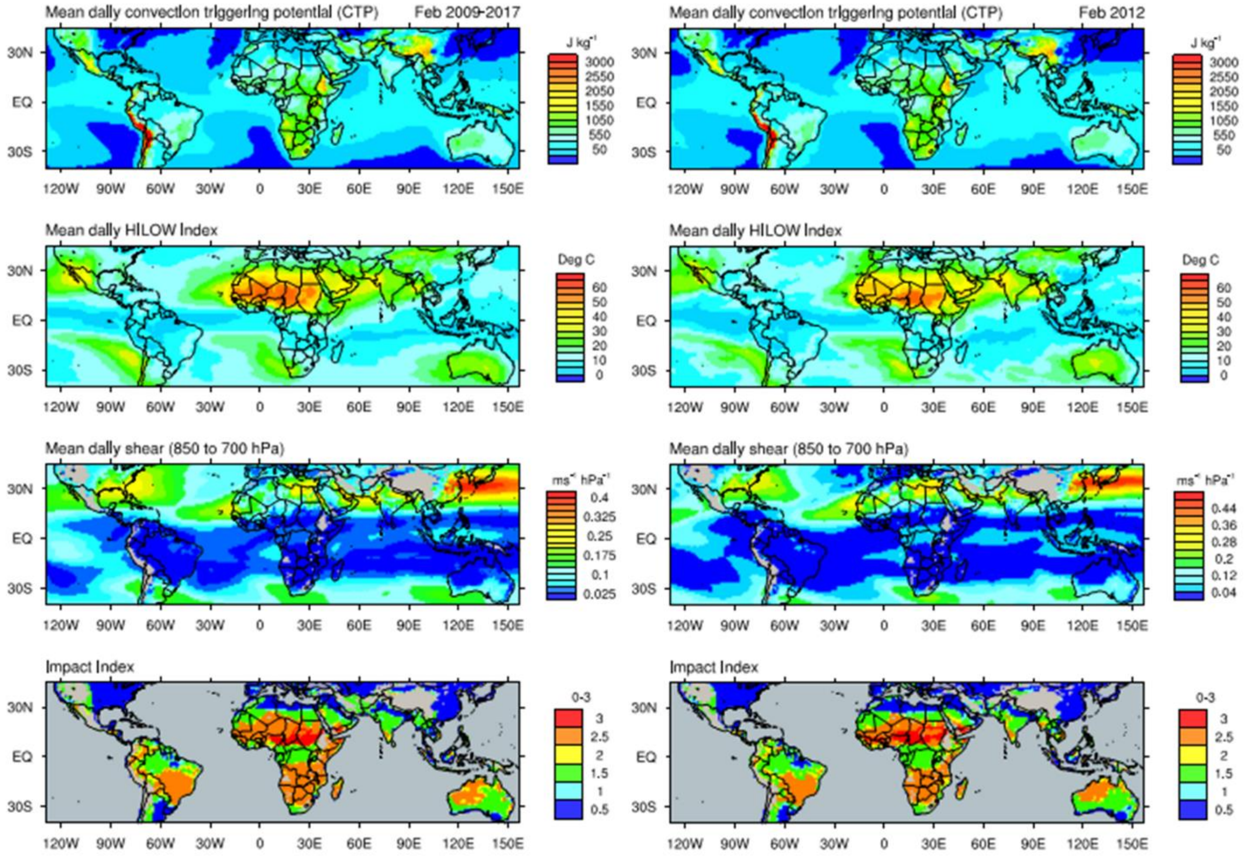

Fig S5. February - monthly mean values of daily means (left panels 2009-2017, right panels 2012 only) for CTP, HIlow, Shear, and GFI.

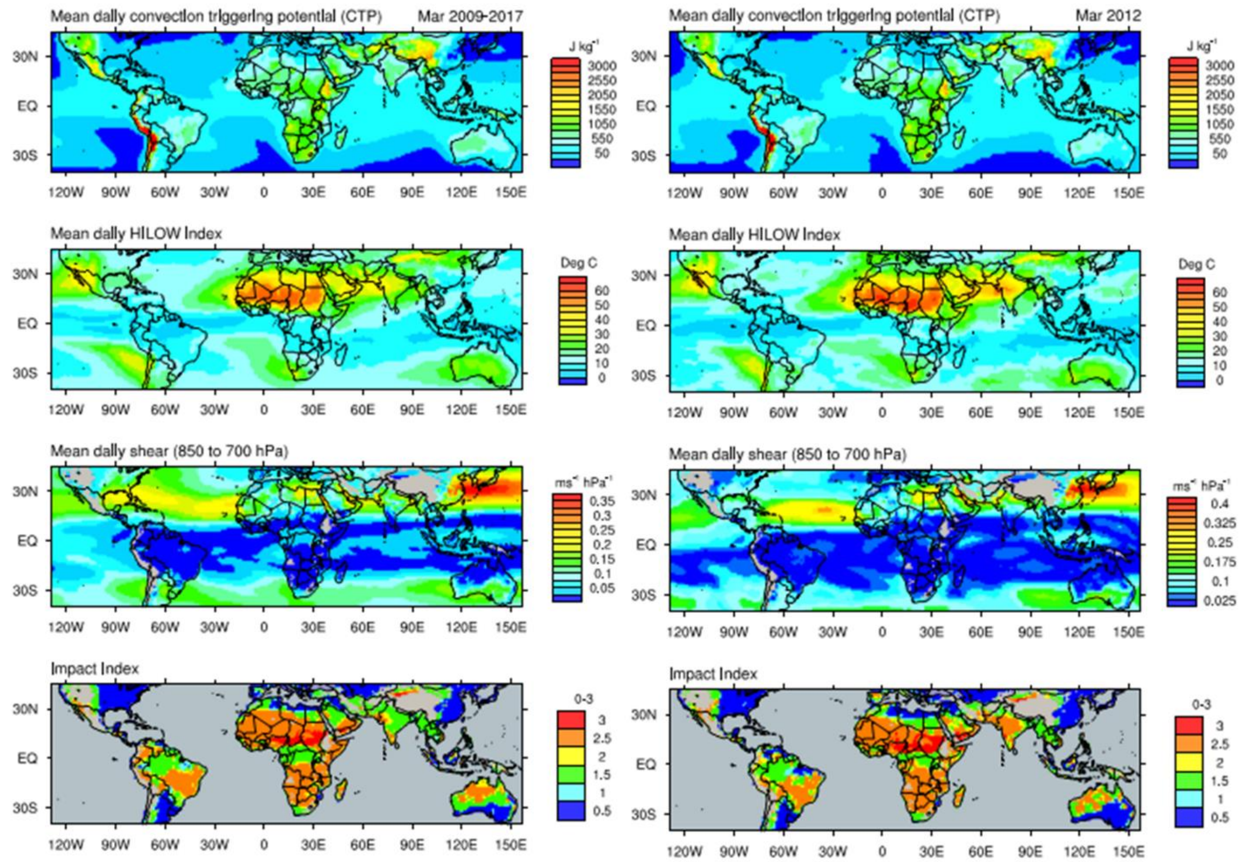

Fig S6. March - monthly mean values of daily means (left panels 2009-2017, right panels 2012 only) for CTP, HIlow, Shear, and GFI.

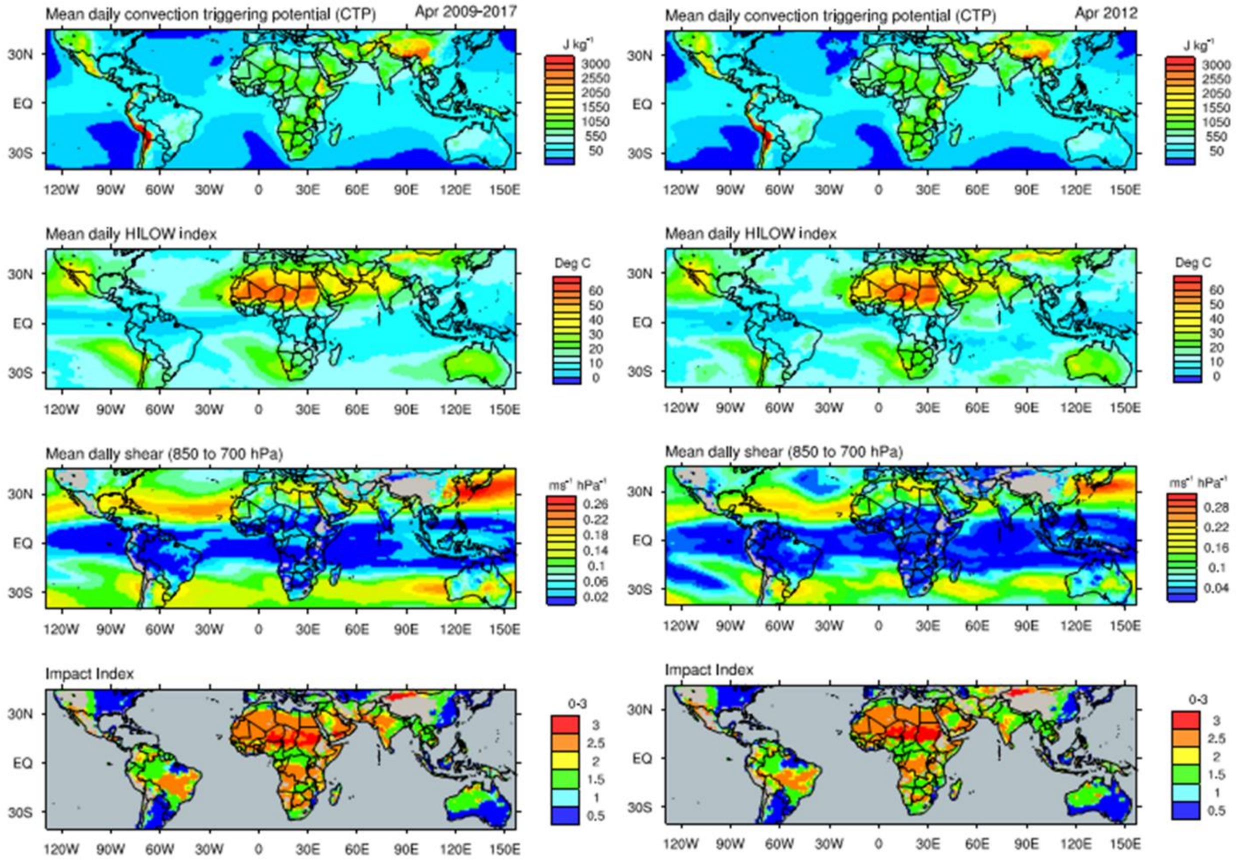

Fig S7. April - monthly mean values of daily means (left panels 2009-2017, right panels 2012 only) for CTP, HIlow, Shear, and GFI.

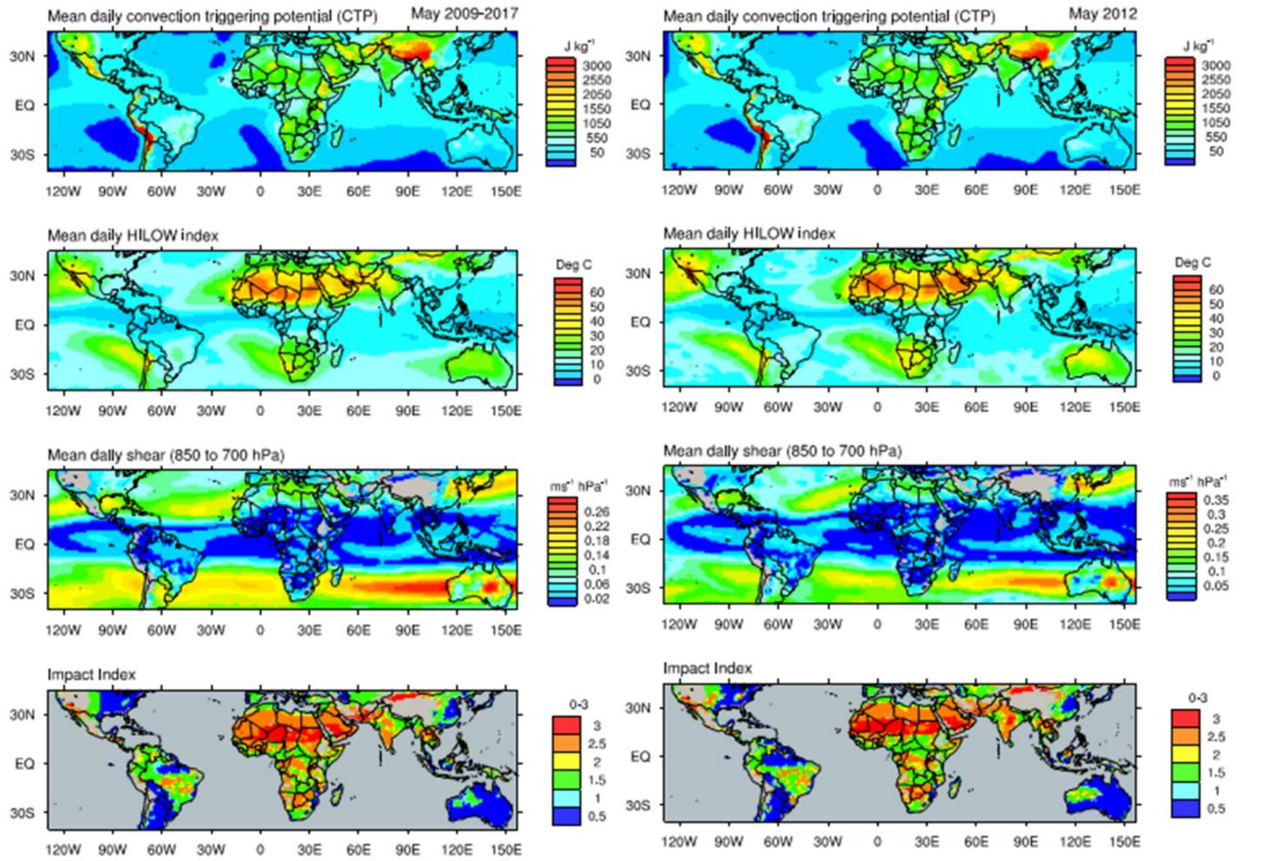

Fig S8. May - monthly mean values of daily means (left panels 2009-2017, right panels 2012 only) for CTP, HIlow, Shear, and GFI.

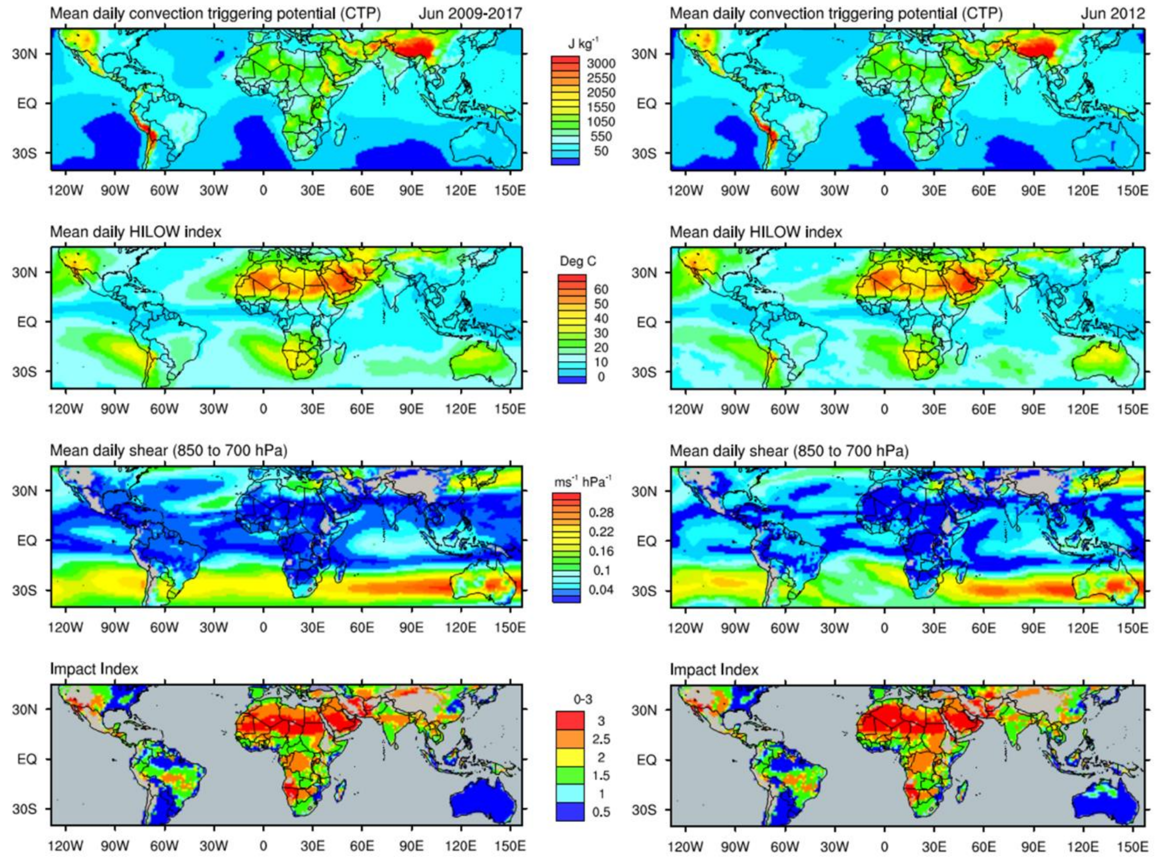

Fig S9. June - monthly mean values of daily means (left panels 2009-2017, right panels 2012 only) for CTP, HIlow, Shear, and GFI.

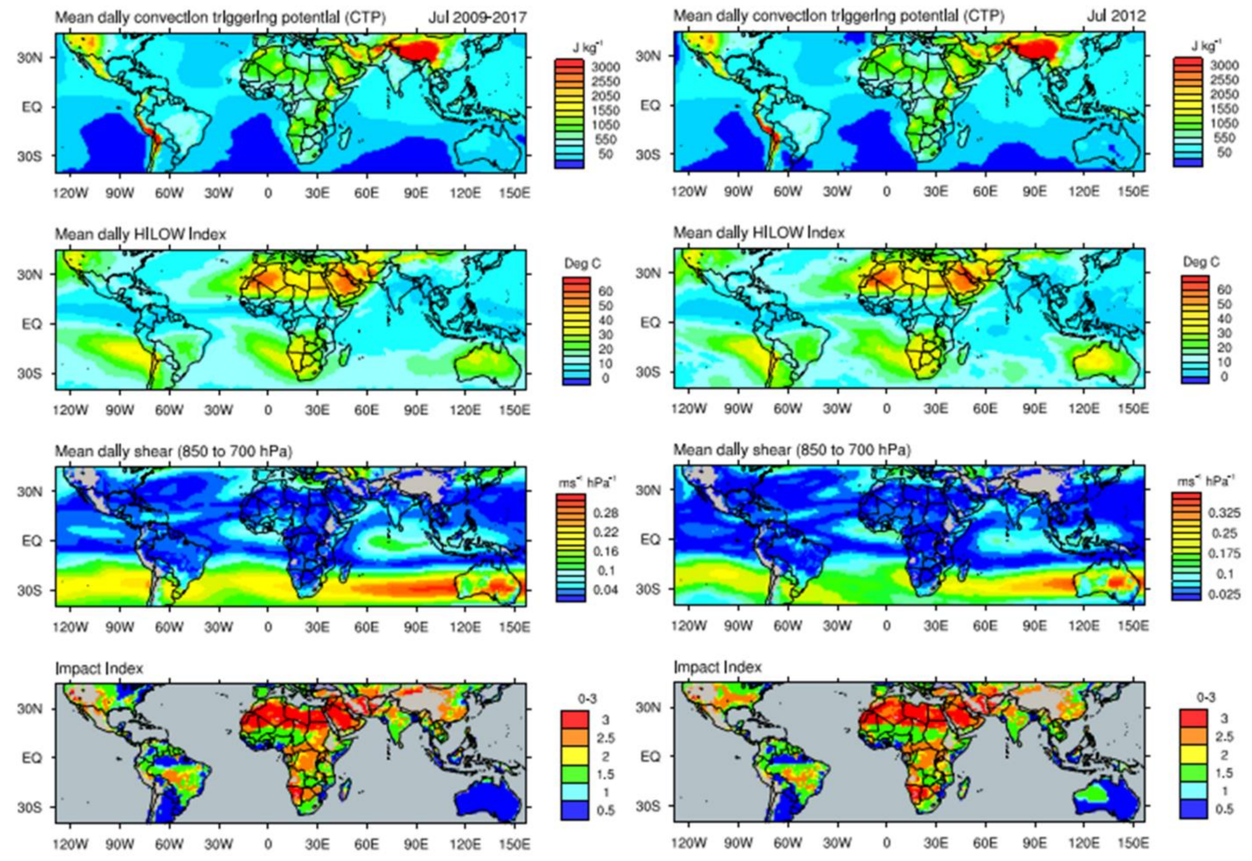

Fig S10. July - monthly mean values of daily means (left panels 2009-2017, right panels 2012 only) for CTP, HIlow, Shear, and GFI.

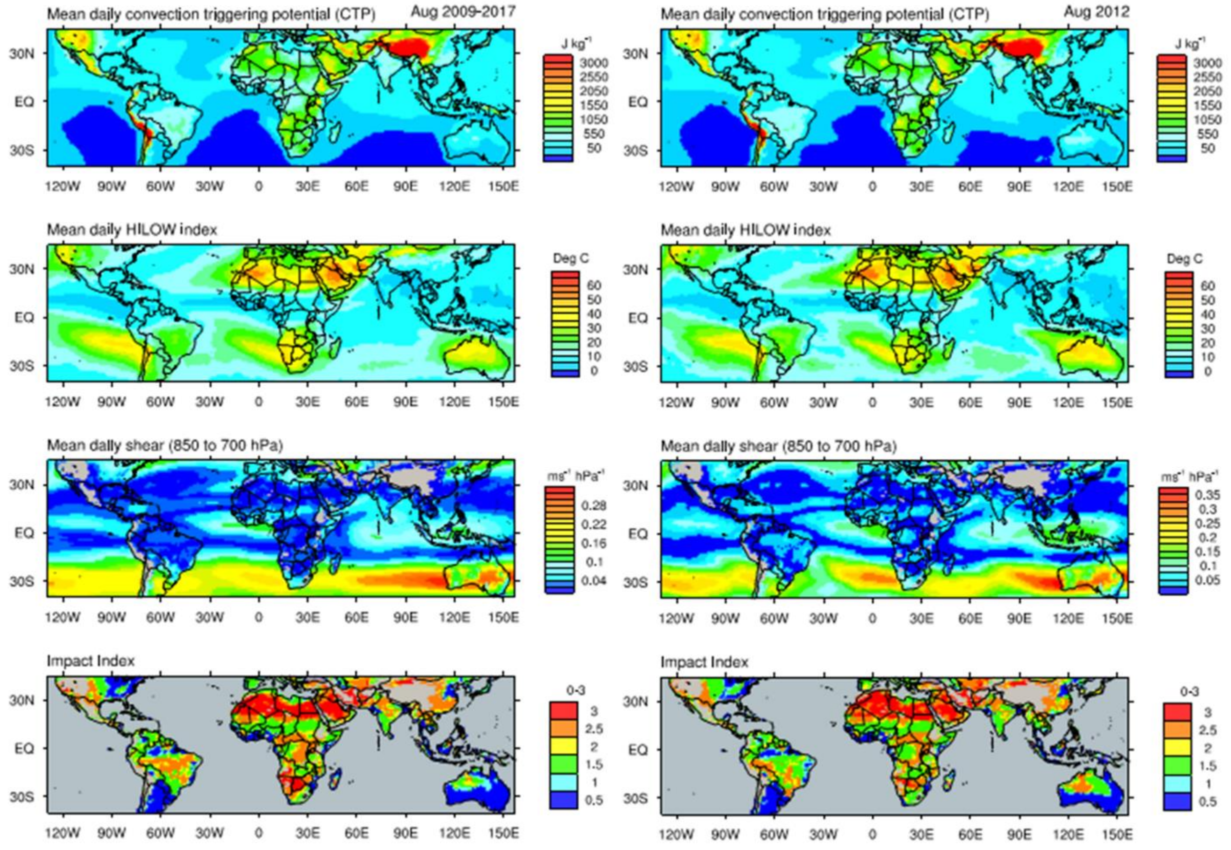

Fig S11. August - monthly mean values of daily means (left panels 2009-2017, right panels 2012 only) for CTP, HIlow, Shear, and GFI.

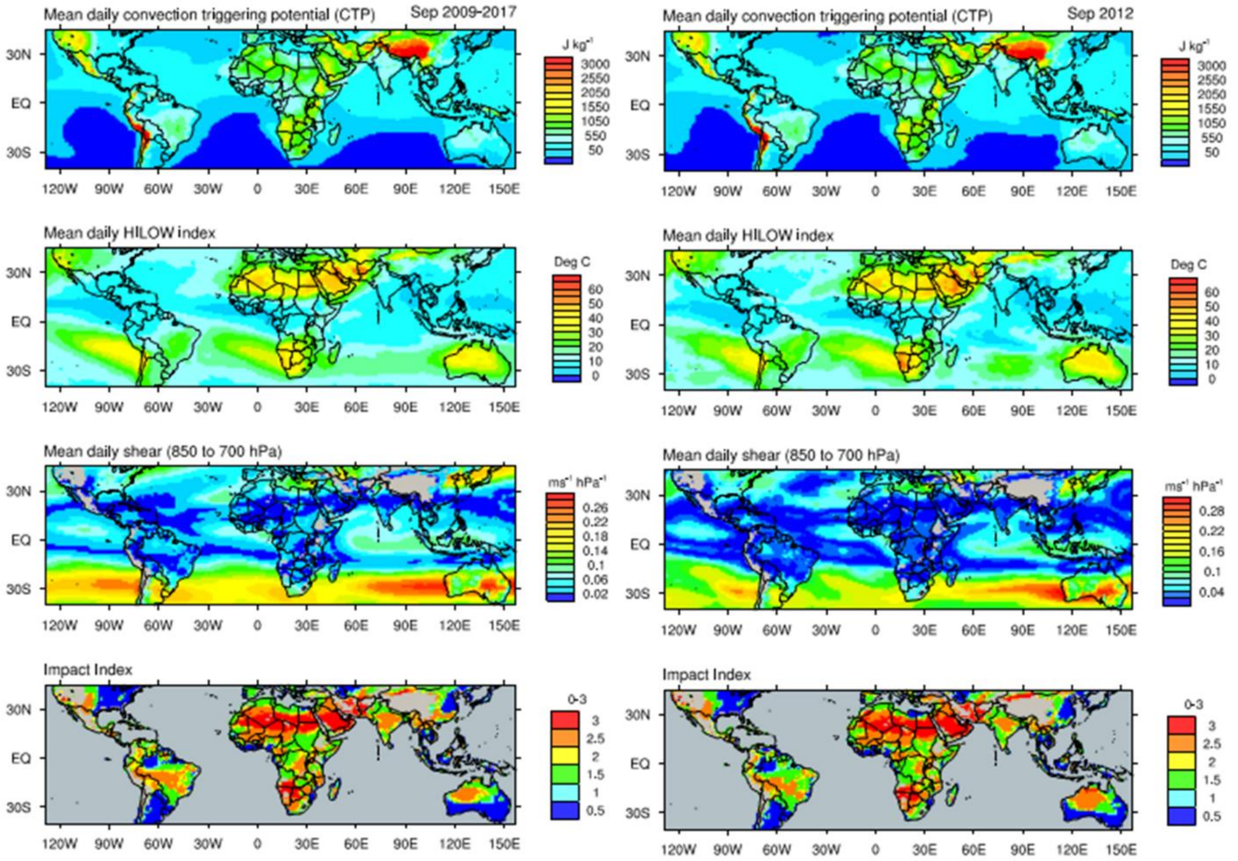

Fig S12. September - monthly mean values of daily means (left panels 2009-2017, right panels 2012 only) for CTP, HIlow, Shear, and GFI.

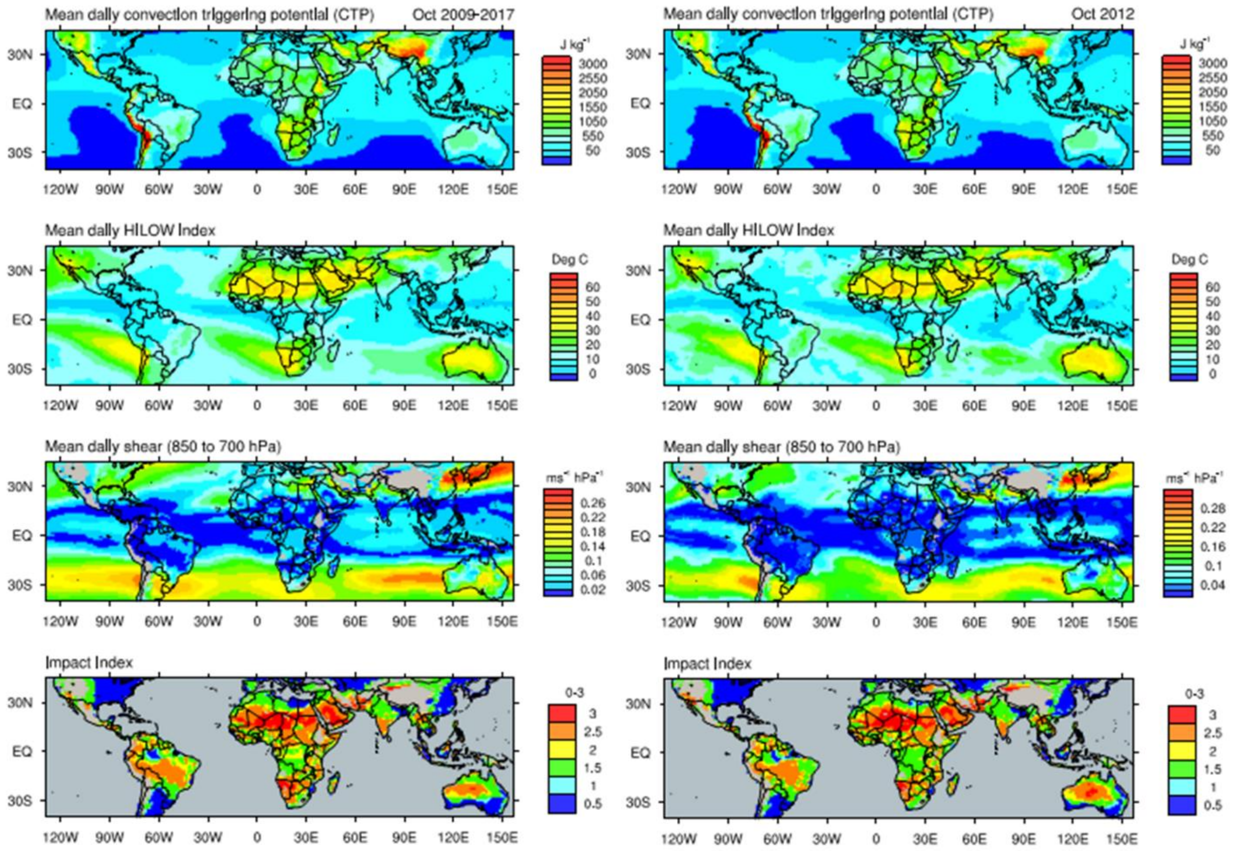

Fig S13. October - monthly mean values of daily means (left panels 2009-2017, right panels 2012 only) for CTP, HIlow, Shear, and GFI.

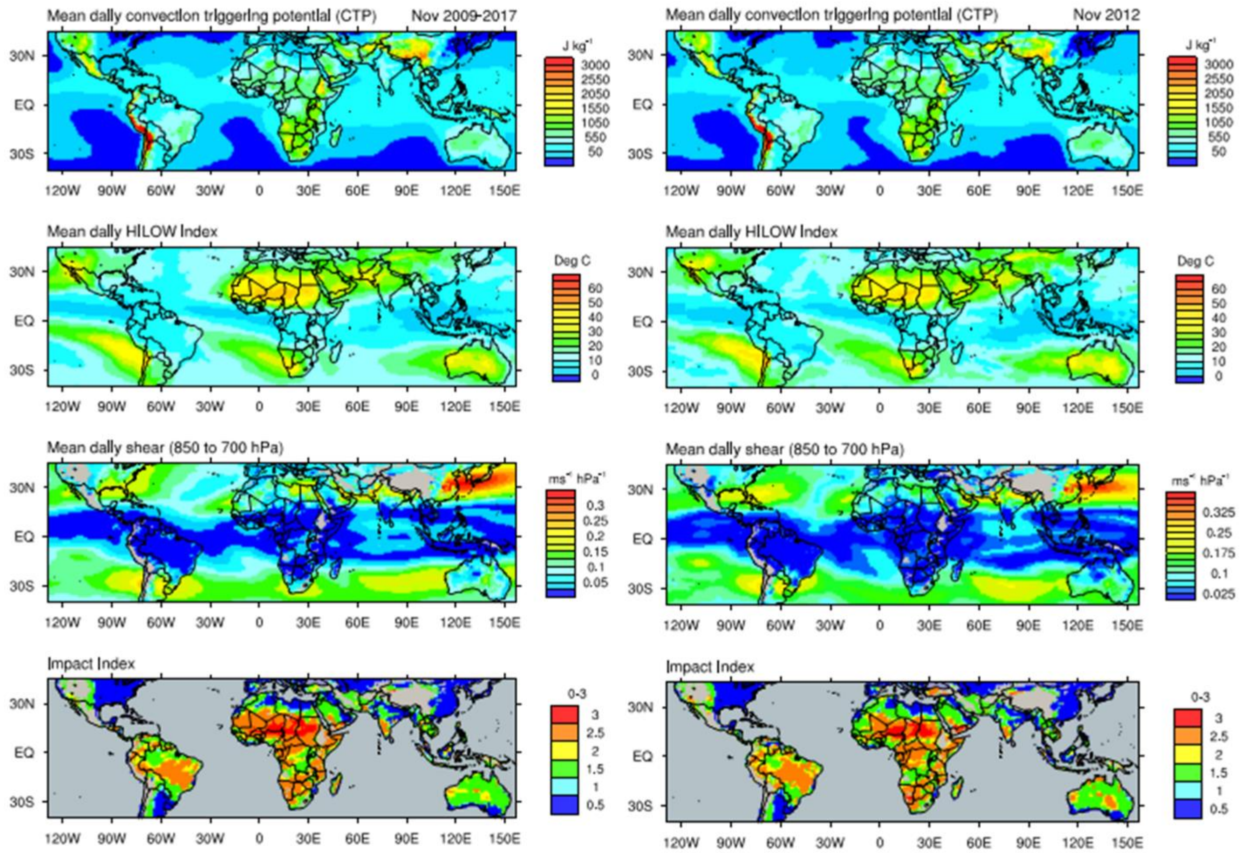

Fig S14. November - monthly mean values of daily means (left panels 2009-2017, right panels 2012 only) for CTP, HIlow, Shear, and GFI.

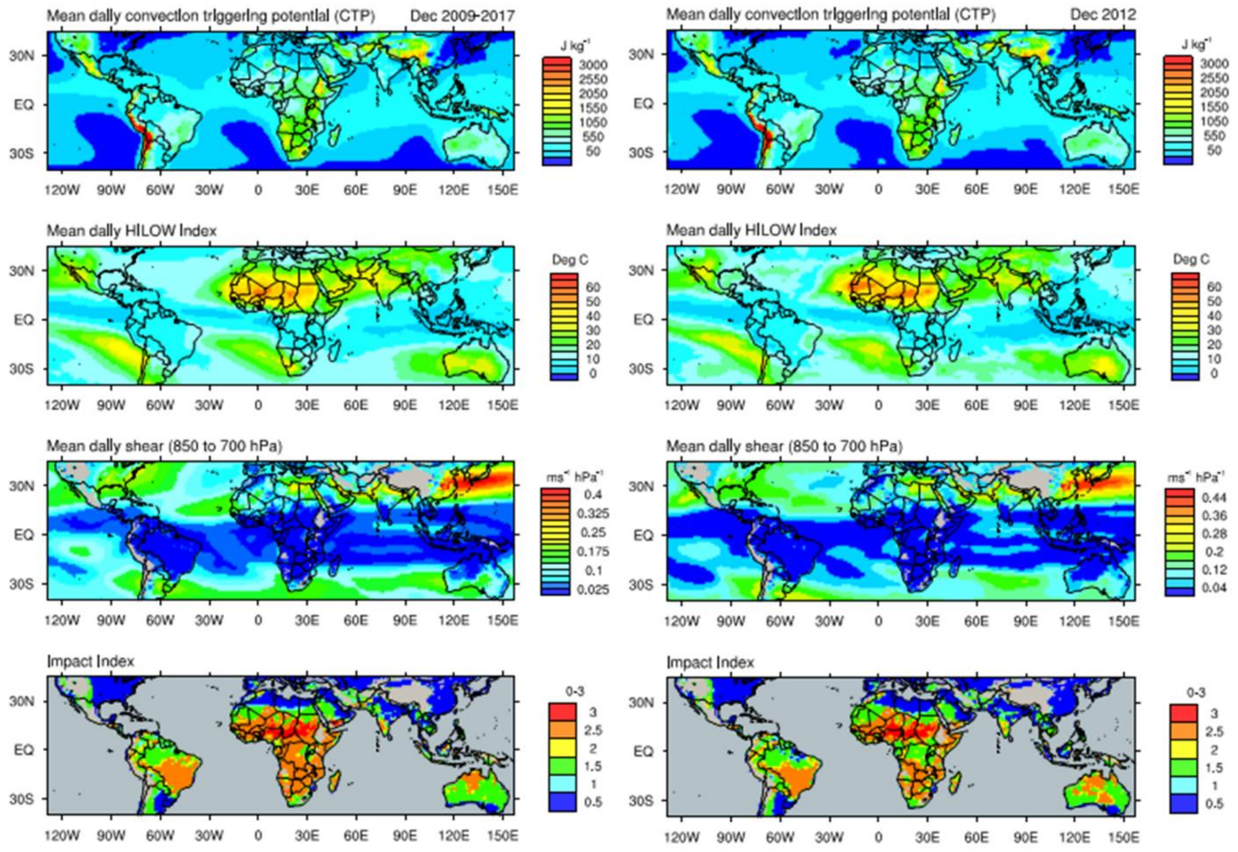

Fig S15. December - monthly mean values of daily means (left panels 2009-2017, right panels 2012 only) for CTP, HIlow, Shear, and GFI.

Apart from small anomalies, 2012 appears to be highly representative of the 9 year (2009-2017) period and consequently that the analysis of 2012 model data is reasonable to use for assessment of GFI thresholds.

Highlight - Land surface-atmosphere feedbacks

Here we demonstrate the plantation impact process chain in more detail, from the land surface up to the upper troposphere. Fig. S16 shows the plantation effect close at the land surface upon heating and pressure/wind deformation, on 30th June 2012 when a CI event with rainfall was observed (up to 9 mm day^{-1} in some grid points around the plantation).

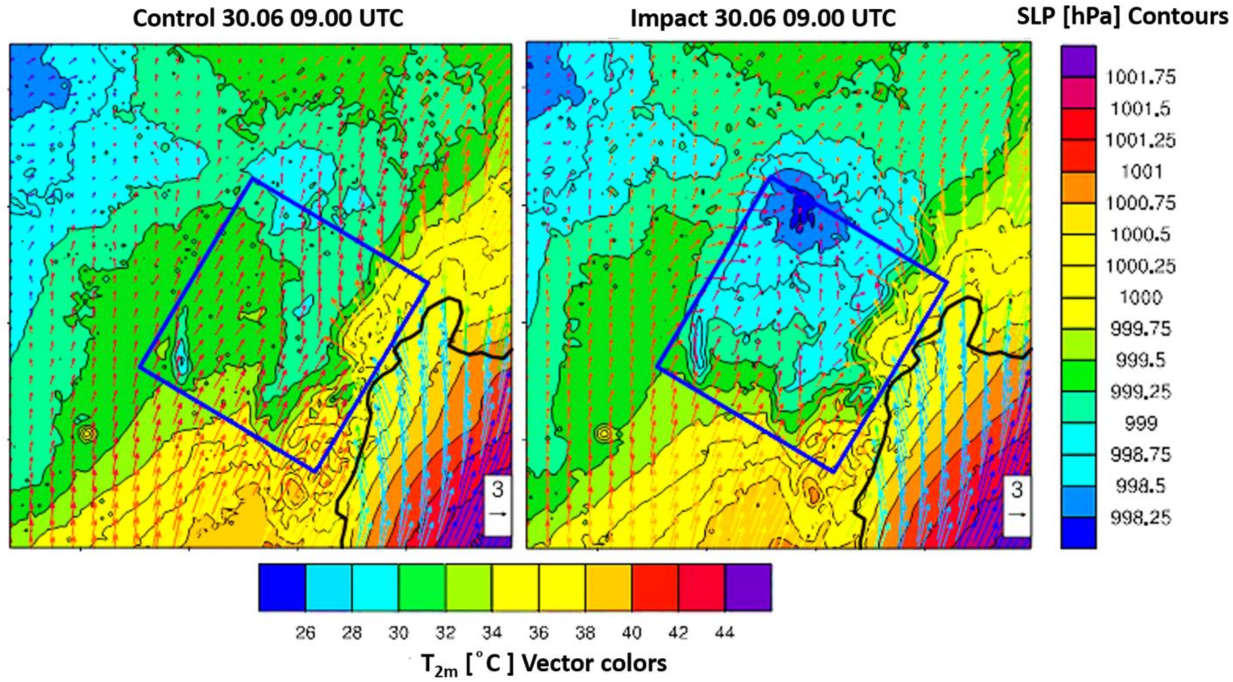

Fig. S16. The impact of a 100 × 100 km coastal plantation in Oman on sea level pressure (SLP, hPa, filled contours), 2m air temperature (T_{2m}, ˚C, vector colors), and 10m wind flows ($\overrightarrow{UV}_{10}$, m s^{-1}, wind vectors, 3m scale arrow) on 30th June 2012 at 9.00 UTC (13.00 LT), as observed in WRF-NOAH. Left panel is the CONTROL run i.e. no plantation, but with plantation outline shown and on the right, the IMPACT run.

There was a clear heat-low air pressure reduction of 1 to 1.5 hPa, and this pressure gradient force together with surface friction led to a convergence of winds toward the leeside of the plantation. Fig. S17 shows the impacts further up in the planetary boundary layer (PBL).

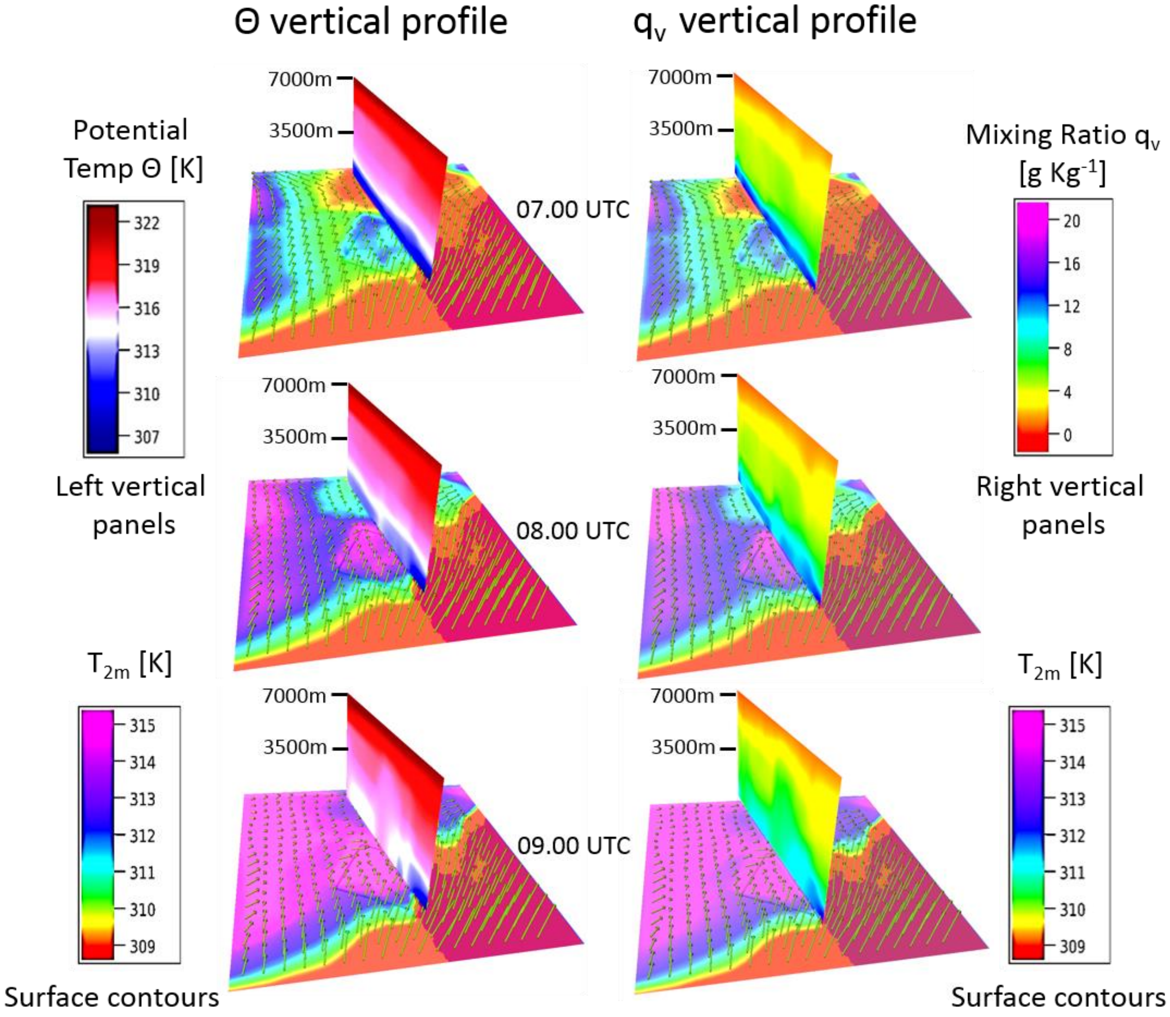

Fig. S17. The impact of the plantation in Oman on 2m air temperature (T_{2m}, K, horizontal planes, filled contours), and 10m wind flows (($\overrightarrow{UV}_{10}$, wind vectors), potential temperature (Θ, K, left-hand vertical profiles), and water vapor mixing ratio (q_v, g kg^{-1}, right-hand vertical profiles) on 30th June 2012 at 7.00, 8.00 and 9.00 UTC (11.00-13.00 LT), as observed in WRF-NOAH Impact run.

We observed clearly that the wind convergence and increased turbulence over the plantation created a strong upward mixing of heat and vapor to altitudes of over 5000 m, higher than the level of free convection. This process of de-stratifying the PBL occurs over the 3 hours between 11.00 and 13.00 local time. See also Movie S1 for a 3D animation of PBL evolution and CI on June 30th 2012.

References for SI reference citations

1. Skamarock, W. C. *et al. A Description of the Advanced Research WRF Version 3*. (2008). https://opensky.ucar.edu/islandora/object/technotes%3A500/datastream/PDF/view DOI: 10.5065/D68S4MVH.

2. Chen, F. & Dudhia, J. Coupling an Advanced Land Surface Hydrology Model with the Penn State NCAR MM5 Modeling System. Part I: Model Implementation and Sensitivity. Mon. Weather Rev. 129 (4), 569–585 (2001). DOI: 10.1175/1520-0493(2001)129<0587:CAALSH>2.0.CO;2

3. ECMWF - European Centre for Medium Range Weather Forecasting - Operational analysis data (0.125˚)

4. Hong, S.-Y., Noh, Y. & Dudhia, J. A New Vertical Diffusion Package with an Explicit Treatment of Entrainment Processes. *Mon. Weather Rev.* **134**, 2318–2341 (2006). DOI: 10.1175/MWR3199.1

5. Dyer, A. J. & Hicks, B. B. Flux-gradient relationships in the constant flux layer. *Q. J. R. Meteorol. Soc.* **96**, 715–721 (1970). DOI: 10.1002/qj.49709641012

6. Paulson, C. A. The Mathematical Representation of Wind Speed and Temperature Profiles in the Unstable Atmospheric Surface Layer. *J. Appl. Meteorol.* **9**, pp. 857–861 (1970). DOI: 10.1175/1520-0450(1970)009<0857:TMROWS>2.0.CO;2

7. Morrison, H. & Gettelman, A. A New Two-Moment Bulk Stratiform Cloud Microphysics Scheme in the Community Atmosphere Model, Version 3 (CAM3). Part I: Description and Numerical Tests. *J. Clim.* **21**, 3642–3659 (2008). DOI: 10.1175/2008JCLI2105.1

8. Iacono, M. J. *et al.*, Radiative forcing by long-lived greenhouse gases: Calculations with the AER radiative transfer models. *J. Geophys. Res. Atmos.* **113**, 2–9 (2008).

9. Mlawer, E. J., Taubman, S. J., Brown, P. D., Iacono, M. J. & Clough, S. A. Radiative transfer for inhomogeneous atmospheres: RRTM, a validated correlated-k model for the longwave. *J. Geophys. Res.* **102**, 16663 (1997). DOI: 10.1029/97JD00237

10. Milovac, J., K. Warrach-Sagi, A. Behrendt, F. Späth, J. Ingwersen, and V. Wulfmeyer, 2016: Investigation of PBL schemes combining the WRF model simulations with scanning water vapor differential absorption lidar measurements. J. Geophys. Res. Atmos. 121 (2), 624–649. DOI:10.1002/2015JD023927

11. Balzarini, A. *et al.* Sensitivity analysis of PBL schemes by comparing WRF model and experimental data. *Geosci. Model Dev. Discuss.* **7**, 6133–6171 (2014). DOI: 10.5194/gmdd-7-6133-2014

12. Chaouch, N., Temimi, M., Weston, M. & Ghedira, H. Sensitivity of the meteorological model WRF-ARW to planetary boundary layer schemes during fog conditions in a coastal arid region. *Atmos. Res.* **187**, 106–127 (2017). DOI: 10.1016/j.atmosres.2016.12.009

13. Cohen, A. E., Cavallo, S. M., Coniglio, M. C. & Brooks, H. E. A Review of Planetary Boundary Layer Parameterization Schemes and Their Sensitivity in Simulating Southeastern U.S. Cold Season Severe Weather Environments. *Weather Forecast.* **30**, 591–612 (2015). DOI: 10.1175/waf-d-14-00105.1

14. Ingwersen, J. *et al.* Comparison of Noah simulations with eddy covariance and soil water measurements at a winter wheat stand. *Agric. For. Meteorol.* **151**, 345–355 (2011). DOI:10.1016/j.agrformet.2010.11.010

15. Ek, M. B. *et al.* Implementation of Noah land surface model advances in the National Centers for Environmental Prediction operational mesoscale Eta model. *J. Geophys. Res. Atmos.* **108**, 2002JD003296 (2003). DOI:10.1029/2002JD003296

16. Srivastava, P. K. *et al.* Performance evaluation of WRF-Noah Land surface model estimated soil moisture for hydrological application: Synergistic evaluation using SMOS retrieved soil moisture. *J. Hydrol.* **529**, 200–212 (2015). DOI:10.1016/J.JHYDROL.2015.07.041

17. Jankov, I. *et al.* An Evaluation of Five ARW-WRF Microphysics Schemes Using Synthetic GOES Imagery for an Atmospheric River Event Affecting the California Coast. *J. Hydrometeorol.* **12**, 618–633 (2011). DOI: 10.1175/2010jhm1282.1

18. Orr, A. *et al.* Sensitivity of simulated summer monsoonal precipitation in Langtang Valley, Himalaya, to cloud microphysics schemes in WRF. *J. Geophys. Res.* **122**, 6298–6318 (2017). DOI: 10.1002/2016JD025801

19. Putnam, B. J., Xue, M., Jung, Y., Zhang, G. & Kong, F. Simulation of Polarimetric Radar Variables from 2013 CAPS Spring Experiment Storm-Scale Ensemble Forecasts and Evaluation of Microphysics Schemes. *Mon. Weather Rev.* **145**, 49–73 (2016). DOI:10.1175/mwr-d-15-0415.1

20. Cintineo, R., Otkin, J. A., Xue, M. & Kong, F. Evaluating the Performance of Planetary Boundary Layer and Cloud Microphysical Parameterization Schemes in Convection-Permitting Ensemble Forecasts Using Synthetic GOES-13 Satellite Observations . *Mon. Weather Rev.* **142**, 163–182 (2013). DOI: 10.1175/mwr-d-13-00143.1

21. Koster, R. D. *et al.*, Regions of Strong Coupling Between Soil Moisture and Precipitation. *Science (80).* **305**, 1138–1140 (2004). DOI: 10.1126/science.1100217

22. Findell, K. L., Gentine, P., Lintner, B. R. & Kerr, C. Probability of afternoon precipitation in eastern United States and Mexico enhanced by high evaporation. *Nat. Geosci.* **4,** 434–439 (2011). DOI: 10.1038/ngeo1174

23. Milovac, J., Ingwersen, J., & Warrach-Sagi, K. Soil texture forcing data for the whole world for the Weather Research and Forecasting (WRF) Model of the University of Hohenheim (UHOH) based on the Harmonized World Soil Database (HWSD) at 30 arc-second horizontal resolution (2014).

24. Branch, O., Warrach-Sagi, K., Wulfmeyer, V., & Cohen, S. Simulation of semi-arid biomass plantations and irrigation using the WRF-NOAH model - a comparison with observations from Israel. *Hydrol. Earth Syst. Sci.* (2014). DOI: 10.5194/hess-18-1761-2014

25. Choudhury B. J. *et al.*, A biophysical process-based estimate of global land surface evaporation using satellite and ancillary data: II. Regional and global patterns of seasonal and annual variations. *J. Hydrol.* **205**, 186–204 (1998). DOI: 10.1016/S0022-1694(97)00147-9

26. Frei, C. Daily precipitation statistics in regional climate models: Evaluation and intercomparison for the European Alps. *J. Geophys. Res.* **108**, 4124 (2003). DOI: 10.1029/2002JD002287

27. Findell, K. & Eltahir, E. Atmospheric controls on soil moisture-boundary layer interactions. Part I: Framework development. *J. Hydrometeorol.*, 552–569 (2003). DOI: 10.1175/1525-7541(2003)004<0552:ACOSML>2.0.CO;2

28. Findell, K. L. Atmospheric controls on soil moisture-boundary layer interactions: Three-dimensional wind effects. J. Geophys. Res. 108, 8385 (2003). DOI: 10.1029/2001JD001515

29. ECMWF - European Centre for Medium Range Weather Forecasting - ERA-5 re-analysis data (0.28125˚)

Caption for Movie S1

Movie still.

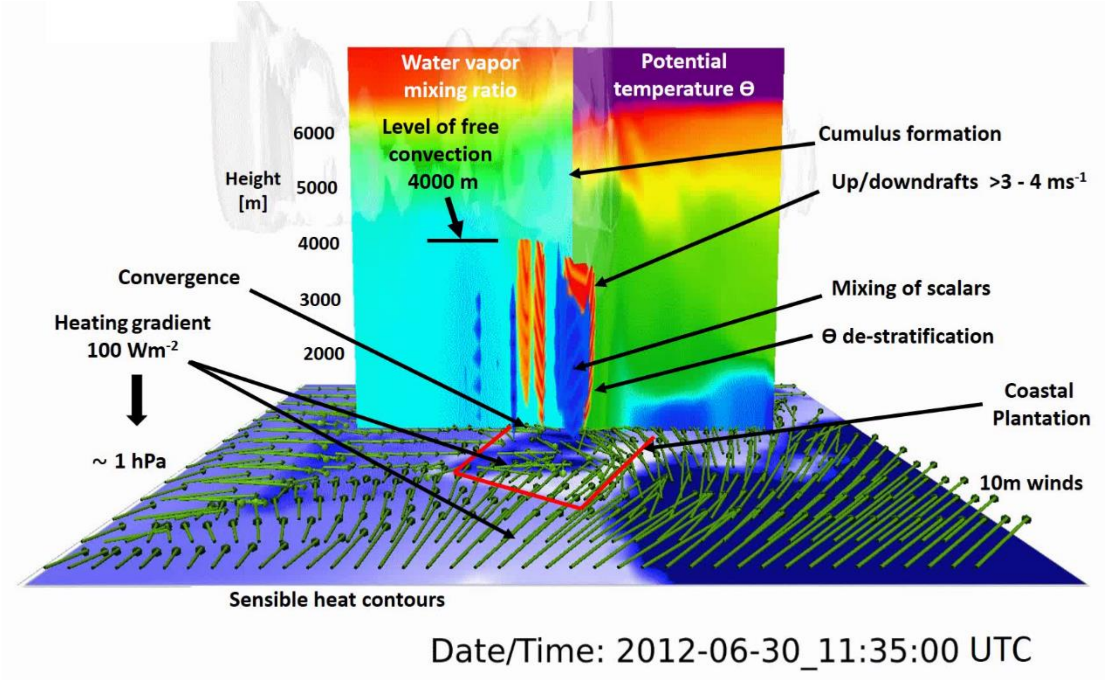

Movie S1. The impact of a plantation on the evolution of the planetary boundary layer on 30th June 2012, on a day when convection was initiated, from 09:00 until 18:00 local time. The animation depicts a 100 × 100 km coastal plantation in central Oman. Prevailing southern monsoonal 10m winds are shown as wind vector arrows, sensible heating is shown on filled base contours, water vapor mixing ratio is shown on the left vertical cross section, and potential temperature on the right vertical cross section. Created in the NCAR/UCAR VAPOR package (Visualization and Analysis Platform for Ocean, Atmosphere, and Solar Research).
